# Preparation of Customizable Ligand-Functionalized Chitin Biopolymer Adsorbents Utilizing the Azide–Alkyne “Click” Reaction for the Adsorption of Aqueous Cu(II)

**DOI:** 10.3390/polym18192450

**Published:** 2026-10-08

**Authors:** Blaine M. Berrington, Reyla Williams, Richard LaDouceur

**Affiliations:** 1 Department of Chemistry and Geochemistry, Montana Technological University, 1300 West Park Street, Butte, MT 59701, USA; rladouceur@mtech.edu; 2 Department of Biomedical Engineering, Worcester Polytechnic Institute, 100 Institute Road, Worcester, MA 01609, USA; rbwilliams@wpi.edu

**Keywords:** chitin, click chemistry, copper-catalyzed azide–alkyne cycloaddition (CuAAC), adsorption, Cu(II), heavy metal removal, biopolymer, water treatment, waste valorization

## Abstract

Chitin is an abundant biopolymer, with 1–3 million tons potentially available annually from shellfish-processing waste, but remains underutilized because its insolubility in conventional solvents makes it difficult to process and functionalize. Herein, a three-step route to chitin adsorbents with interchangeable ligands is reported. Highly acetylated chitin was tosylated and converted to the C-6 azide, and amine- and hydroxyl-terminated ligands were attached to this common intermediate by the copper-catalyzed azide–alkyne cycloaddition (CuAAC) “click” reaction, yielding water-insoluble derivatives. Cu(II) adsorption to the adsorbents was evaluated using inductively coupled plasma optical emission spectrometry (ICP-OES analysis). Under standard conditions (pH 6.0, 4 h contact), amine- and alcohol-chitin adsorbed 30.1 ± 1.3 and 22.3 ± 1.9 mg Cu g^−1^, respectively, approximately 4.9- and 3.6-fold more than unmodified chitin. Isotherm data were well described by the Langmuir model, with apparent capacities of 37.4 and 25.7 mg g^−1^ at 4 h contact. The adsorbents could be regenerated with 7% (*v*/*v*) HCl, retaining 46% and 42% of their initial uptake after four cycles. The differences in derivatives’ uptakes are attributable to the identity of the grafted ligand, illustrating how the click route allows chitin adsorbents to be tailored through the choice of alkyne.

## 1. Introduction

Chitin is a naturally occurring and abundant polysaccharide biopolymer consisting of *N*-acetylglucosamine monomeric units. In the environment, chitin is found as the principal structural component of fungal cell walls and of all arthropod exoskeletons and shells. As such, chitin is the second most abundant biopolymer on the planet, second only to cellulose [1]. It is estimated that 1–3 million tons of chitin could be made available every year from shellfish waste alone, which is otherwise discarded by the fishing industry [2]. However, the ubiquity of chitin stands in stark contrast to the utilization of this renewable natural resource. It is estimated that 75% of total shellfish weight ends up as waste [3], and between 6 and 8 million tons of shrimp, crab, and lobster shells are produced by fishing operations annually [4]. Despite its abundance, chitin finds few practical commercial or industrial uses, a major aspect of its underutilization as a resource [5]. The lack of resource utilization for chitin is a two-fold problem stemming from a lack of applications and from the difficulty of processing chitin [6]. While there is a growing pool of applications for chitin and it has become a subject of interest, there is still no significant demand for the raw material on a commercial scale [2]. The other aspect of chitin’s underutilization lies in the difficulty of processing raw chitin, hindering its development as a material. Alpha-chitin is the predominant crystalline polymorph of chitin derived from shrimp shells. This form of chitin exhibits antiparallel crystalline sheet packing, resulting in exceptionally strong intermolecular and intramolecular hydrogen bonding of the crystalline sheets [7]. Chitin, especially alpha-chitin with a high degree of acetylation, and therefore a high degree of hydrogen bonding, exhibits strong chemical resistance and robust mechanical properties, making it difficult to process into secondary materials because it resists solubilization and physical processing methods such as heat molding [7,8,9].

Substantial effort has been invested in developing use cases for chitin as a resource. Chitin has been explored for many applications, including drug delivery [10,11], cell-culture scaffolds [12,13,14], catalyst supports [15,16,17], and components of composite materials [18,19,20,21]. Another potential use of chitin is wastewater treatment. A derivative of chitin, chitosan, has been explored extensively as a chelating adsorption material but dissolves in dilute acid, which limits its use in acidic effluents without crosslinking [22,23]; chitin itself, however, has received little attention for this application. The few attempts at utilizing raw chitin as a heavy-metal adsorbent have fallen short, yielding poor adsorption uptakes and reinforcing the need to modify chitin to improve its adsorption capabilities [24,25,26]. The reason is primarily that the chitin nitrogens are acetylated and lack the chelating ability of deacetylated chitosan [27]. However, chitin has potential as a support polymer for functional surface chemistries tailored to various adsorption applications [24,28]. One such possibility is designed functional groups for the adsorption of heavy-metal pollutants in water. Chitin could provide the water-insoluble backbone for such functionalities, a strong, chemically resistant foundation onto which the adsorbing functionalities are grafted. Such functionalization has been explored in some capacity, but typically with chitosan or with chitin of a lower degree of acetylation than demonstrated herein [28,29,30,31,32].

To functionalize chitin, a method is needed that modifies its functional groups selectively and efficiently. The click chemistry approach introduced by Sharpless and co-workers is well suited to this purpose [33,34,35]. Its hallmark reaction, the copper-catalyzed azide–alkyne cycloaddition (CuAAC) [33], is efficient, highly selective, and tolerant of a wide range of solvent and pH conditions [35,36], and it has been used widely to modify polysaccharides, including cellulose [37,38] and chitosan [30,32]. Because different alkynes can be coupled to the same azide-bearing backbone, CuAAC in principle allows ligands to be interchanged to suit a given adsorption target. Its application to highly acetylated chitin has been limited. Chen et al. converted the C-6 hydroxyl of chitin to the tosylate and then the azide, attached β-cyclodextrin by CuAAC, and deacetylated the product to a chitosan derivative [39]. Kritchenkov et al. introduced azide groups by ring-opening of 2-(azidomethyl)oxirane and prepared water-soluble triazole chitin derivatives for catalytic and antibacterial applications [40], as well as a chelating derivative used as a support for a Pd(II) catalyst [41].

As for the pollutant targets, heavy metals pose substantial risks to human health and to the general health of terrestrial ecosystems [42]. One of the most common pollutants, copper, has been found to be harmful to human organs at elevated concentrations [43,44,45] and has been linked to the development of certain cancers [46,47]. Copper is also exceptionally toxic to aquatic life [48]. Copper represents a proof-of-concept adsorption target for highly customizable surface functionalities. Adsorption of copper for wastewater treatment provides a framework for demonstrating the tunability of the functionalization, which could then be applied to a broader swath of heavy-metal pollutants.

The present work differs from the previously reported methods in its products and purpose. An analogous C-6 tosylate–azide route of Chen et al. [39] is used here, but the products are retained as highly acetylated, water-insoluble chitin rather than deacetylated or solubilized, since insolubility over the working pH range is a prerequisite for a reusable adsorbent. The method has been modified to accommodate highly acetylated chitin. Small functional alkynes of propargylamine and propargyl alcohol are used to introduce amine- and hydroxyl-terminated ligands, and both are attached to a single batch of azide-chitin, so that the effect of ligand identity can be compared on a common backbone. To our knowledge, the resulting triazole-tethered aminomethyl and hydroxymethyl chitin derivatives have not been reported previously, and CuAAC-functionalized highly acetylated chitin has not been evaluated as an adsorbent for metal ions in water, although CuAAC has been used to prepare cellulose-based Cu(II) adsorbents [38]. The Cu(II) adsorption of the two derivatives is compared here with that of unmodified chitin. Because the coupling step uses a copper catalyst, the possible contribution of residual catalyst to the measured Cu(II) uptake is also examined. The present work extends our earlier study of unmodified chitin, in which chitin flakes were physically expanded into higher-surface-area foams to increase Cu(II) uptake without altering their chemistry [26]; here, chitin is instead modified chemically, so that adsorption can be tuned through the grafted ligand, and no results from that study are reused. This work provides a basis for chitin adsorbents with interchangeable ligands and a route toward added-value use of chitin-rich waste.

## 2. Materials and Methods

### 2.1. Materials

Chitin flakes derived from shrimp shells (>98% acetylation) were purchased from Sigma-Aldrich. *p*-Toluenesulfonyl chloride (>98.5%), sodium azide (>98%), dimethyl sulfoxide (DMSO, >98%), dimethylacetamide (DMAc), lithium chloride (>99%), sodium ascorbate (>99%), copper sulfate (anhydrous, >99%), propargylamine (>98%), propargyl alcohol (>98%), methanol (>98%), reagent alcohol, diethyl ether (anhydrous, >99%), acetone (>98%), and triethylamine (>99%) were purchased from Fisher Scientific and used without further purification. Copper(II) nitrate hemipentahydrate from Sigma-Aldrich (>99%) was used to prepare all Cu(II) solutions.

### 2.2. Synthesis of Functionalized Chitin Adsorbents

**Scheme 1:**
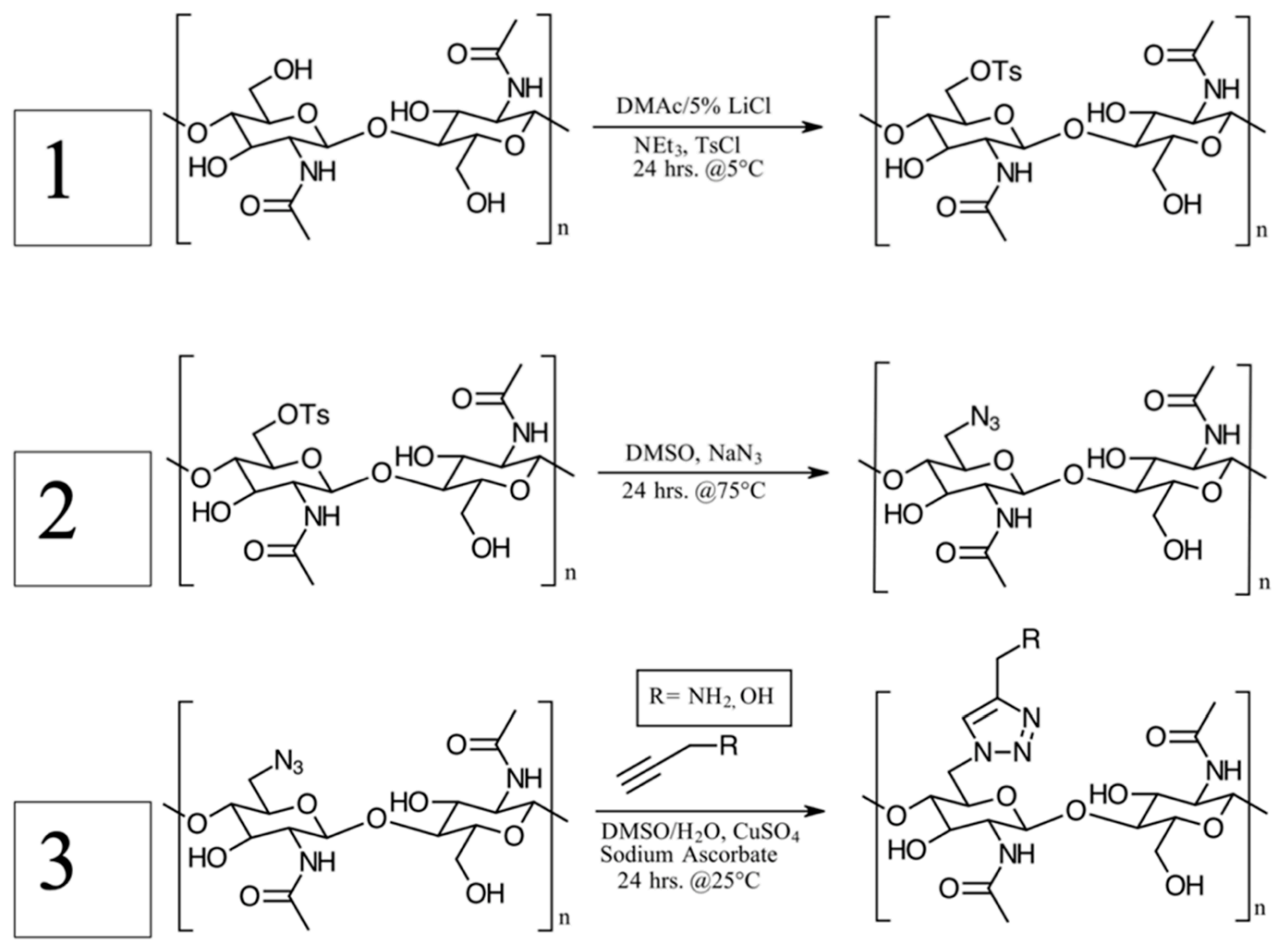
Synthetic procedure for the customizable click functionalization of highly acetylated chitin. The protocol comprises (**1**) tosylation of the C-6 hydroxyl group, (**2**) nucleophilic substitution of the tosylate to form the azide derivative, and (**3**) copper-catalyzed azide–alkyne cycloaddition (CuAAC, “click”) coupling of the azide with the corresponding alkyne ligand.

The overall three-step synthetic route, comprising tosylation, azidation, and CuAAC “click” coupling, is outlined in Scheme 1.

#### 2.2.1. Tosylation of the Carbon-6 (C-6) Position of Chitin

To 1.0 g of (>98% acetylated) chitin flakes, 9.8684 g of lithium chloride was added, and the mixture was dried for 24 h at 90 °C in an oven. To the lithium chloride and chitin mixture, 200 mL of anhydrous DMAc was added, and the slurry was stirred at 65 °C for 24 h. The solution was then cooled to room temperature, and 8.5 mL of triethylamine (NEt_3_) was added. Subsequently, the solution was cooled to 0 °C on a Stir-Kool^®^ cold plate, and 10.046 g of *p*-toluenesulfonyl chloride dissolved in 25 mL of anhydrous DMAc was added dropwise. The reaction was stirred between 0 and 5 °C for 24 h. The tosyl-chitin was recovered by precipitating the reaction solution in acetone, filtering, washing with acetone, and washing with deionized water (DI water). The tosyl-chitin was then freeze-dried for 24 h to yield a beige solid.

#### 2.2.2. Azidation of C-6 Tosyl-Chitin

A mixture of 1.0 g of tosyl-chitin and 0.83 g of sodium azide was combined, and 50 mL of DMSO was added. The solution was heated at 75 °C overnight. Following the heating period, the yellow-brown colloid was added to 25.0 mL of hot reagent alcohol and filtered. The filtered solid was washed with hot 25 mL portions of reagent alcohol and dried under vacuum to yield a light beige solid. The yield was 97%.

#### 2.2.3. Preparation of the C-6 Amine-Functionalized Chitin Adsorbent

**Figure 1:**
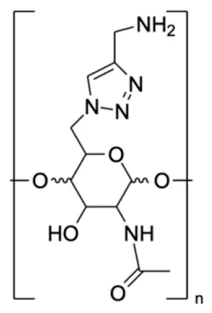
Structure of the amine-chitin derivative prepared by copper-catalyzed azide–alkyne cycloaddition (CuAAC) of azide-functionalized chitin with propargylamine.

A suspension of 0.50 g of azide-chitin in 10 mL of DMSO was stirred and allowed to swell for 30 min. Following the swelling induction period, 0.20 mL of propargylamine was added. To the reaction mixture, 0.1783 g of sodium ascorbate in 1.0 mL of DI water and 0.04790 g of anhydrous copper sulfate in 1.0 mL of DI water were added. The reaction mixture was stirred overnight. The product was then filtered, washed with DI water, washed with 7% (*v*/*v*) HCl solution, washed again with DI water, and dried under vacuum to yield a light brown solid (Figure 1). The yield was 98%. Before use, the product was soaked in deionized water adjusted to pH 7 for 4 h; the water was then replaced and the soak repeated, and the second soak solution was analyzed for copper by ICP-OES.

#### 2.2.4. Preparation of the C-6 Alcohol-Functionalized Chitin Adsorbent

**Figure 2:**
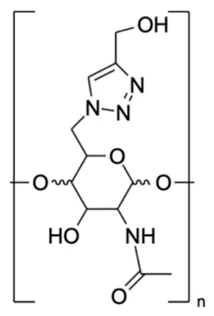
Structure of the alcohol-chitin derivative prepared by copper-catalyzed azide–alkyne cycloaddition (CuAAC) of azide-functionalized chitin with propargyl alcohol.

A suspension of 0.50 g of azide-chitin in 10 mL of DMSO was stirred and allowed to swell for 30 min. Following the swelling induction period, 0.25 mL of propargyl alcohol was added. To the reaction mixture, 0.1813 g of sodium ascorbate in 1.0 mL of DI water and 0.04801 g of anhydrous copper sulfate in 1.0 mL of DI water were added. The reaction mixture was stirred overnight. The product was then filtered, washed with DI water, washed with 7% (*v*/*v*) HCl solution, washed again with DI water, and dried under vacuum to yield a light brown solid (Figure 2). The yield was 97%. Before use, the product was soaked in deionized water adjusted to pH 7 for 4 h; the water was then replaced and the soak repeated, and the second soak solution was analyzed for copper by ICP-OES.

### 2.3. Characterization

Diffuse reflectance infrared Fourier transform spectroscopy (DRIFTS) was carried out on a Shimadzu IRTracer-100 spectrometer with a Praying Mantis attachment over the range 700–4000 cm^−1^ at a resolution of 4 cm^−1^. Solid-state ^13^C nuclear magnetic resonance (NMR) spectroscopy was carried out by a third party at the California Institute of Technology using a Bruker DSX-500 at 8 kHz with 1.0 ms contact times. Sample morphology was analyzed with a TESCAN Mira scanning electron microscope (SEM). Thermal decomposition profiles were obtained by thermogravimetric analysis (TGA) on a TA Instruments Q50 with a ramp rate of 5 °C min^−1^ to a maximum temperature of 450 °C. Cu(II) concentrations in the adsorption experiments were determined by inductively coupled plasma optical emission spectrometry (ICP-OES) on a Thermo Scientific iCAP 6500 series instrument at the Cu 324.754 and 327.395 nm emission lines. Samples were filtered through 0.45 µm syringe filters and diluted 10-fold in 2% HNO_3_ before analysis. The instrument was calibrated with standards of 0.1, 1, 5, 10, 25, and 50 mg L^−1^ (r^2^ ≥ 0.997), and quality control comprised initial and continuing calibration blanks and verification standards (ICB, ICV, CCB, and CCV) and method blanks, with the CCV and CCB analyzed after every tenth sample. The relative standard deviation of replicate readings was 0.5%, and the detection limit for Cu was 0.004 mg L^−1^. X-ray diffraction (XRD) patterns were collected on a Shimadzu XRD-7000 with Co Kα radiation (λ = 0.179 nm) over 2θ = 5–50° in 0.02° steps. Zeta potentials were recorded with an Anton Paar SurPASS 3 analyzer over the pH range 3.0–7.0.

### 2.4. Adsorption Experiments

Cu(II) solutions were prepared from copper nitrate in deionized water at the concentrations stated for each experiment. All adsorption experiments were performed in triplicate, each replicate being an independent vessel charged with a separately weighed portion of adsorbent and a freshly prepared aliquot of Cu(II) solution. Every experiment was accompanied by adsorbent-free control vessels prepared and handled identically, to quantify any loss of dissolved Cu(II) to precipitation, vessel walls, or the separation procedure. Results are reported as the mean ± one standard deviation of the three replicates.

*Standard batch adsorption.* An adsorbent dose of 1700 mg L^−1^ (25.0 mg of dry adsorbent in 15.0 mL of solution) was equilibrated with 200 mg L^−1^ Cu(II) at pH 6.0 and 25 °C for 4 h on a shaker table at 250 rpm. Following equilibration, the adsorbent was sedimented by centrifugation and the supernatant was filtered through a 0.45 µm syringe filter prior to analysis. Post-adsorption Cu(II) concentrations were determined by ICP-OES as described in Section 2.3.

*pH control.* Solution pH was adjusted to the target value before the adsorbent was added, allowed to equilibrate with the adsorbent for approximately 30 min, and then readjusted to the target value with concentrated NaOH or HNO_3_. The pH of each vessel was measured again at the end of the contact period and remained at the target value in all experiments.

The adsorption capacity (Q_e_) was determined according to Equation (1):

(1) $$Q_{e}\;=\;\frac{(C_{0}\;-\;C_{e})V}{m}$$

where Q_e_ is the adsorption capacity of Cu(II) (mg Cu g^−1^), C_0_ is the initial Cu(II) concentration (mg L^−1^), C_e_ is the Cu(II) concentration after contact (mg L^−1^), m is the dry adsorbent mass (g), and V is the solution volume (L).

*Contact time and equilibration.* Sorption kinetics were determined using independent batch vessels rather than serial sampling: for each contact time, three separate vessels containing 85.0 mg of dry adsorbent in 50.0 mL of 200 mg L^−1^ Cu(II) solution (1700 mg L^−1^ dose, pH 6.0, 25 °C, 250 rpm) were prepared and agitated in parallel. At the designated time each vessel was removed from the shaker, the adsorbent was sedimented by centrifugation, and a single 1.0 mL aliquot of the filtered supernatant was withdrawn for ICP-OES analysis; no vessel was sampled more than once. Vessels were sampled at 30, 60, 90, 120, 150, 180, 240, and 300 min. Because each vessel contributes a single measurement and the aliquot is withdrawn after the contact period has ended, the solution volume is constant throughout the experiment and Equation (2) applies with V as defined for Equation (1):

(2) $$Q_{t}\;=\;\frac{(C_{0}\;-\;C_{t})V}{m}$$

where C_t_ is the Cu(II) concentration (mg L^−1^) at time t and the remaining variables are as defined for Equation (1).

*Extended contact.* An additional experiment of the same design was carried out at 24 and 36 h to characterize uptake beyond the 4 h batch period. For amine-chitin, uptake was 30.8 ± 3.1 mg g^−1^ at 24 h and 36.6 ± 6.1 mg g^−1^ at 36 h, against 28.3 ± 2.4 mg g^−1^ at 4 h; for alcohol-chitin, 20.9 ± 3.4 and 24.3 ± 5.1 mg g^−1^ against 19.1 ± 2.2 mg g^−1^; and for unmodified chitin, 6.5 ± 1.4 and 5.6 ± 2.2 mg g^−1^ against 5.4 ± 2.1 mg g^−1^. No extended-contact value differed significantly from the corresponding 4 h value (*p* > 0.1, Welch’s *t*-test), although the point estimates for both derivatives were higher at 24 and 36 h, and differences of that size cannot be excluded at this level of replication. A contact time of 4 h was adopted as the standard operational condition for all pH, dose, isotherm, and regeneration experiments, extended contact affording no statistically resolvable additional uptake while being impractical for routine batch work. Uptakes reported under these conditions (Q_e_) are accordingly uptakes at 4 h contact.

*Effect of pH.* Batch adsorptions were carried out over pH 3.0–6.0 using 25.0 mg of dry adsorbent in 15.0 mL of 200 mg L^−1^ Cu(II) solution at 25 °C for 4 h, with pH controlled as described above. At pH 7.0 a precipitate formed in the adsorbent-free controls, and adsorption was not measured at this pH.

*Effect of adsorbent dose.* The effect of adsorbent dose was assessed over the range 100–2700 mg L^−1^ using 200 mg L^−1^ Cu(II) solution at pH 6.0 and 25 °C, in a solution volume of 15.0 mL.

*Statistical analysis.* Pairwise comparisons were made with Welch’s *t*-test (α = 0.05) using the means and standard deviations of triplicate measurements. Means and standard deviations for all adsorption experiments are provided in Tables S3–S6.

### 2.5. Regeneration Studies

Following adsorption under the standard conditions (Section 2.4), the adsorbent was recovered by filtration, washed with 7% (*v*/*v*) HCl to desorb Cu(II), rinsed with deionized water, and dried under vacuum overnight. The recovered adsorbent was then used for a further adsorption under the same conditions (200 mg L^−1^ Cu(II), pH 6.0, 25 °C, 4 h), with pH controlled as described in Section 2.4. The procedure was repeated to give an initial adsorption followed by four adsorption–desorption cycles, each in triplicate. Uptake in each cycle was calculated from Equation (1) using the mass of adsorbent recovered at the end of the preceding cycle, and capacity retention was expressed as a percentage of the uptake in the initial cycle, with uncertainties propagated from the replicate standard deviations.

## 3. Results and Discussion

### 3.1. Characterization of the Chitin Derivatives

**Figure 3:**
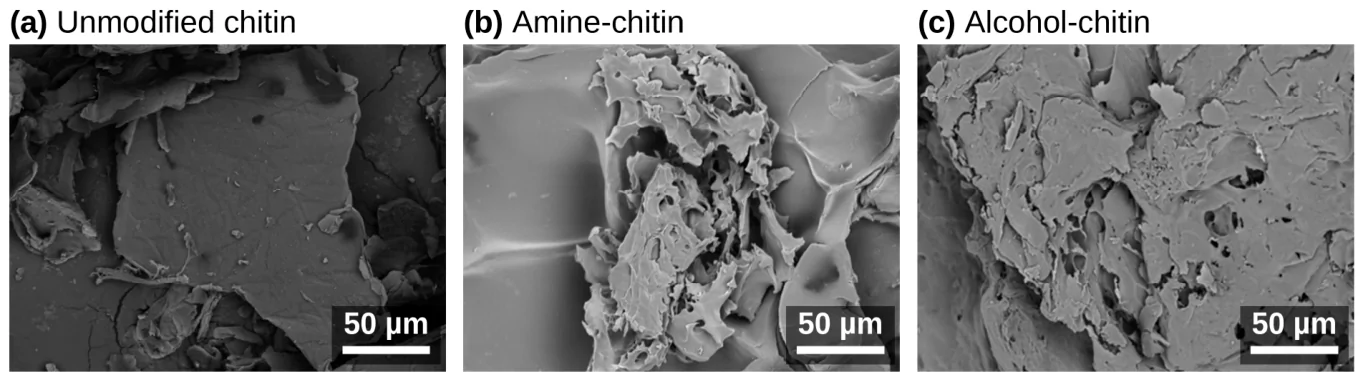
Scanning electron microscopy (SEM) images of (**a**) unmodified chitin, (**b**) amine-chitin, and (**c**) alcohol-chitin, shown at the same scale (249 × 187 µm field; scale bars, 50 µm). Images were acquired on a TESCAN MIRA3 at ×1.1 k magnification, at 15.0 kV with the backscattered-electron (BSE) detector (**a**) and at 10.0 kV with combined BSE and secondary-electron detectors (**b**,**c**). The unmodified flakes show smooth, layered surfaces; both derivatives show a rougher topography.

SEM images of unmodified chitin, amine-chitin, and alcohol-chitin are shown in Figure 3. The unmodified flakes present smooth, layered surfaces, whereas both derivatives show slight delamination of the lamellar structure and a rougher surface topography, consistent with the loss of crystalline order observed by XRD. These observations are qualitative: specific surface area was not measured, and the morphological changes are not invoked to explain the differences in Cu(II) uptake between the materials.

**Figure 4:**
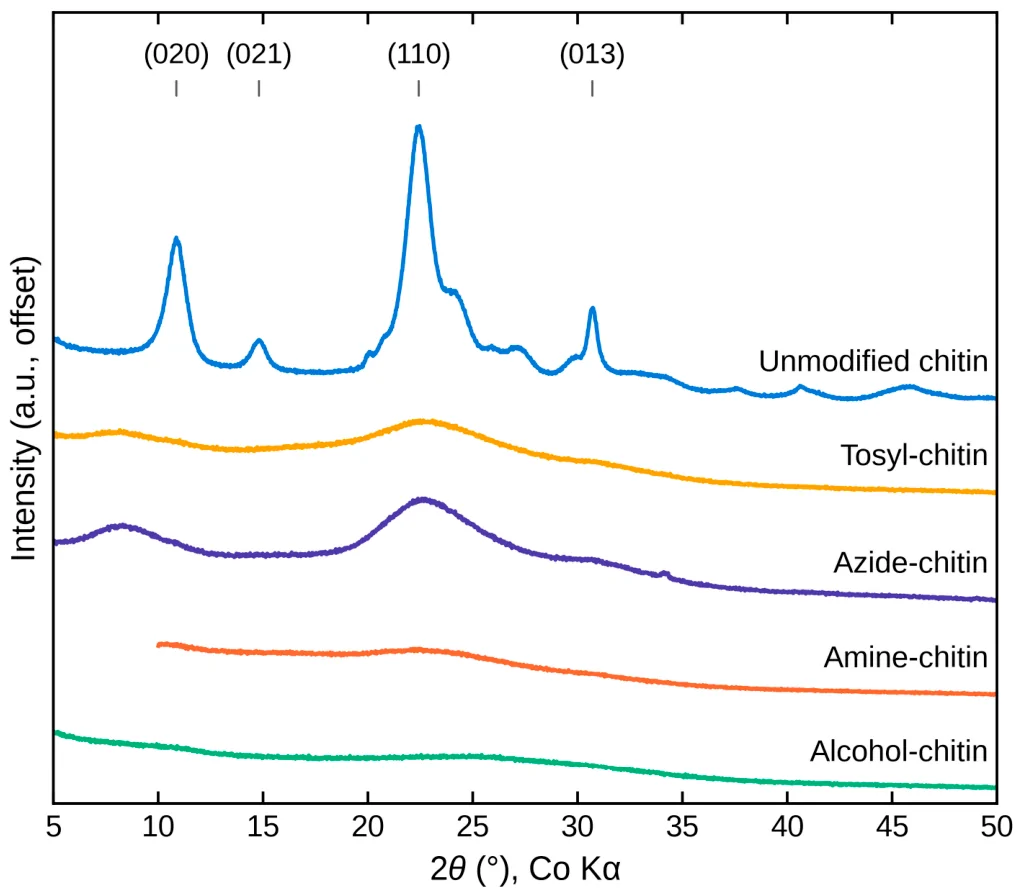
X-ray diffraction (XRD) patterns (Co Kα radiation, λ = 0.179 nm) of unmodified chitin, tosyl-chitin, azide-chitin, amine-chitin, and alcohol-chitin, plotted on a common intensity scale and offset vertically for clarity. The principal α-chitin reflections are indexed on the unmodified-chitin pattern; the amine-chitin pattern was recorded from 2*θ* = 10°. Full patterns with the (110) peak fits are given in Figure S2.

The disruption of the crystal structure seen in the morphology of the derivatives is reinforced by their XRD patterns (Figure 4; full patterns in Figure S2). Unmodified chitin shows the reflections characteristic of α-chitin at 2θ = 10.9, 14.8, 22.4, and 30.7° (Co Kα), which broaden and lose intensity on modification. Two measures of this change are given in Table 1. The crystallinity index, CrI = [(I_110_ − I_am_)/I_110_] × 100 [49], where I_110_ is the maximum intensity of the (110) reflection near 22.4° and I_am_ the intensity minimum near 18°, fell from 83% for unmodified chitin to 25% on tosylation, recovered partially to 42% on azidation, and fell to 8% and 4% for amine- and alcohol-chitin, respectively. The apparent crystallite size normal to the (110) plane was estimated from the Scherrer equation [50,51] (Equation (3)) with K = 0.9 and λ = 0.179 nm:

(3) $$\mathrm{Crystallite}\;\mathrm{size}\;(\mathrm{nm})\;=\;\frac{K\lambda }{\beta \;\cos\;\theta }$$

where K is the shape factor, λ is the X-ray wavelength (nm), β is the full width at half maximum (radians), and θ is the Bragg angle. The full width at half maximum of the (110) reflection was obtained by fitting a pseudo-Voigt profile together with the neighboring reflection near 24.2°, a broad amorphous contribution, and a linear background over 2θ = 16–28.5°. Instrumental broadening was not subtracted, so the values in Table 1 are apparent sizes and represent lower bounds. The crystallite size decreased from 6.9 nm for unmodified chitin to 2.5 and 2.6 nm for tosyl- and azide-chitin; for amine- and alcohol-chitin the (110) reflection was too weak for a reliable estimate. The principal loss of crystalline order therefore occurs on tosylation, and the click products are essentially X-ray amorphous. A consequence of this transition is the disruption of the hydrogen bonding within the α-chitin sheets that imparts much of its characteristic strength [29].

**Figure 5:**
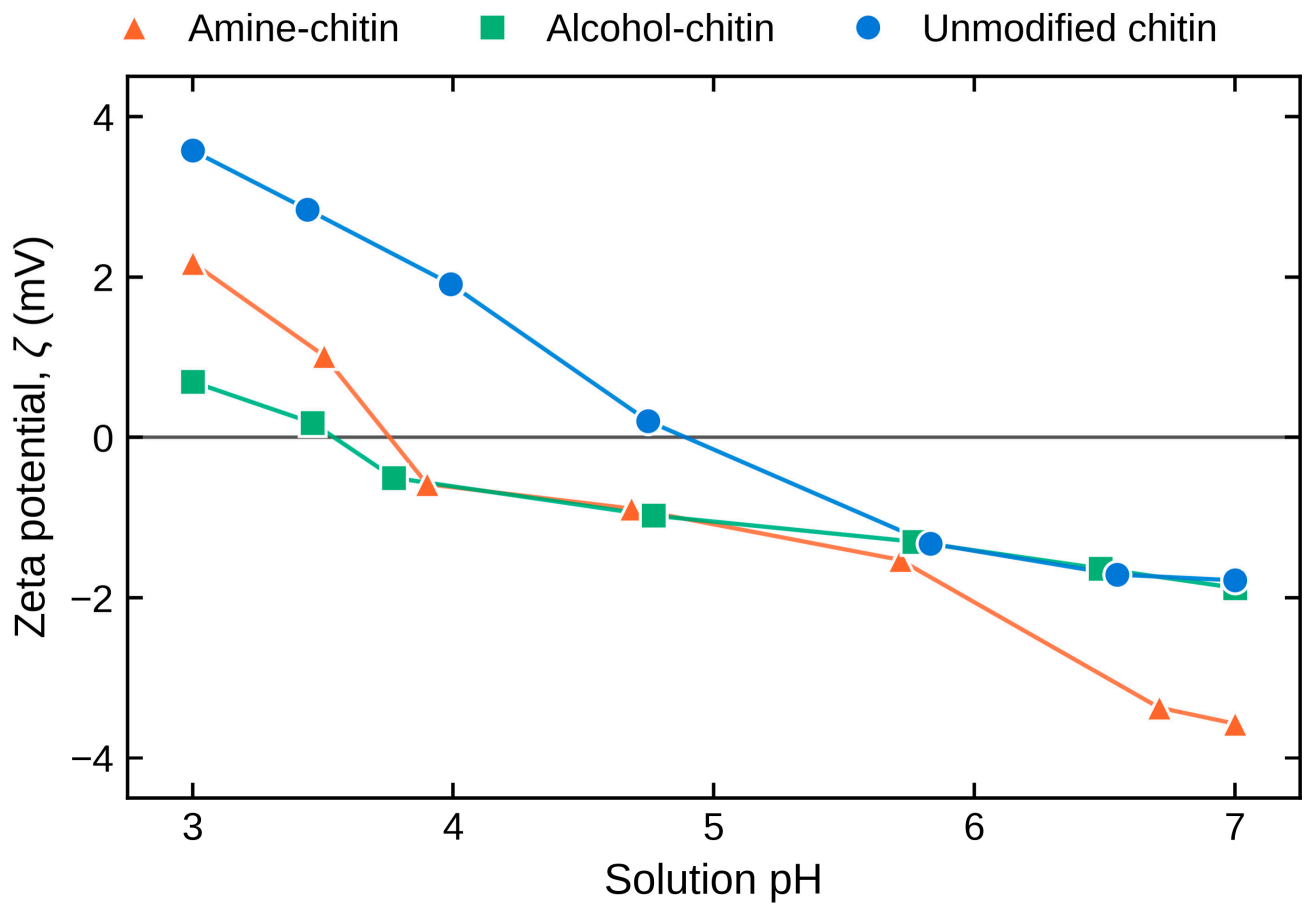
Zeta potential (*ζ*) of amine-chitin, alcohol-chitin, and unmodified chitin as a function of solution pH (pH 3.0–7.0), measured with an Anton Paar SurPASS 3 analyzer. Lines are guides to the eye; the horizontal line marks *ζ* = 0.

Sample surface charges were determined over pH 3.0–7.0 by zeta-potential analysis (Figure 5). The zeta potential of raw unmodified chitin flakes is positive at pH < 4.9, that of amine-chitin at pH < 3.8, and that of alcohol-chitin at pH < 3.5. The surface charges of both functionalized derivatives are decreased by the introduction of the triazole moieties and the amine and alcohol functionalities, respectively, corroborated by similar zeta-potential trends for triazole moieties described by Yin et al. [38]. The shift of surface charge to negative at lower pH for the functionalized chitins relative to unmodified chitin is more conducive to the adsorption of cationic species. At the standard adsorption pH of 6.0, all three materials carry a small negative surface charge, approximately −2.1 mV for amine-chitin and −1.4 mV for both alcohol-chitin and unmodified chitin (interpolated between the measured points in Figure 5).

**Figure 6:**
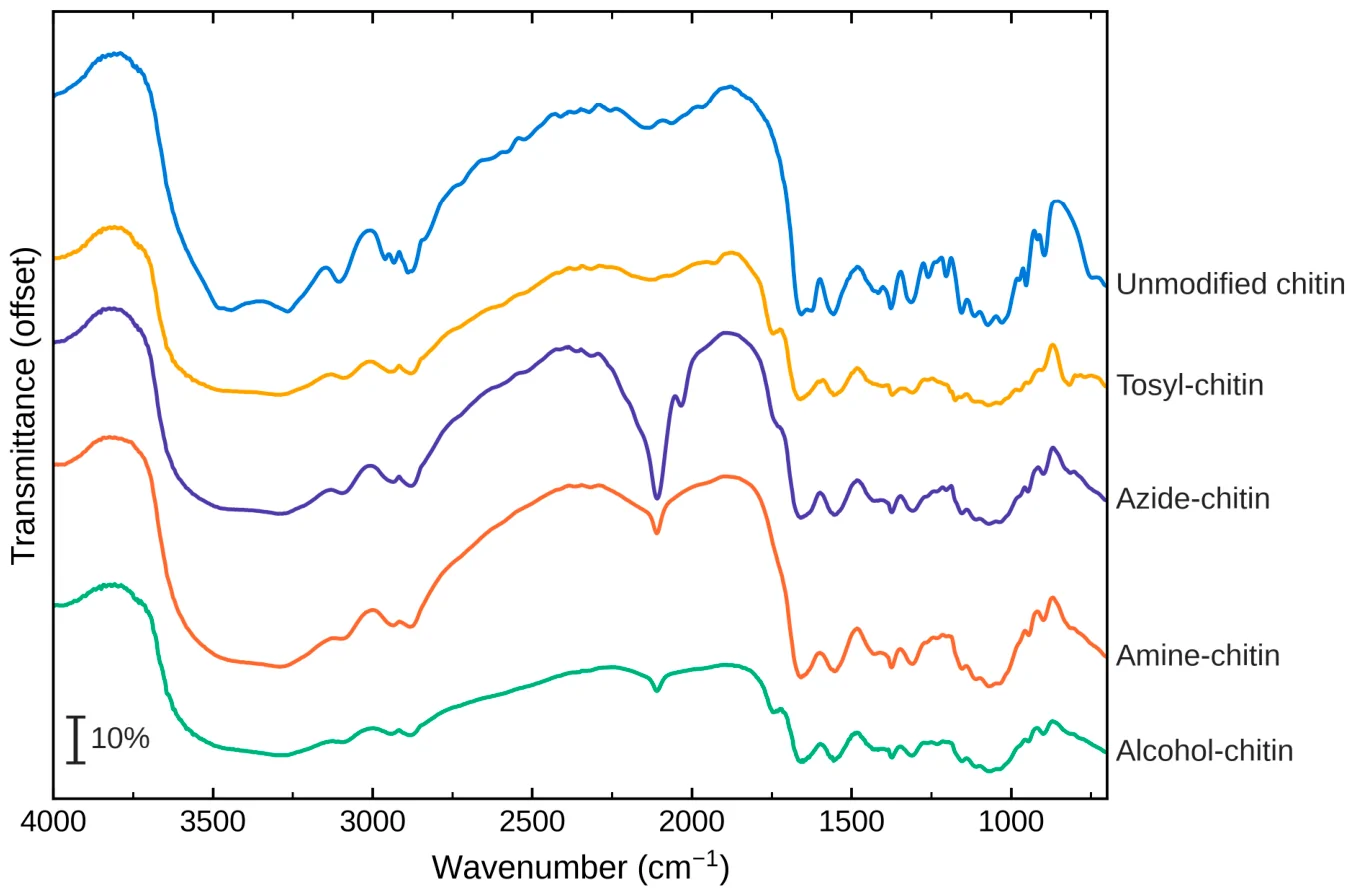
Diffuse reflectance infrared Fourier transform spectroscopy (DRIFTS) spectra of unmodified chitin, tosyl-chitin, azide-chitin, amine-chitin, and alcohol-chitin (top to bottom), recorded over 700–4000 cm^−1^ at 4 cm^−1^ resolution and offset vertically for clarity; the scale bar represents 10% transmittance.

DRIFTS spectra of raw chitin, the tosyl- and azide-chitin intermediates, and the amine- and alcohol-chitin adsorbents are shown in Figure 6. For unmodified chitin, the consistent peaks of interest include the bands at 3200–3500 cm^−1^ (overlapping N–H and O–H stretches), 1655 cm^−1^ (C=O amide stretch), 1110 cm^−1^ (C–O secondary alcohol stretch), 1060 cm^−1^ (C–O primary alcohol stretch), and 1010 cm^−1^ (C–O pyranosidic ether stretch). Preservation of the 1110 cm^−1^ band in all samples indicates that modification is isolated to the C-6 hydroxyl. Preservation of the 1655 cm^−1^ band shows that the *N-acetyl* group is conserved, and the persistent 1010 cm^−1^ band indicates that the glycosidic linkages are also conserved. Tosyl-chitin’s characteristic signals at 800 cm^−1^ (C–O–S stretch) and 1170 cm^−1^ (S=O stretch) mark conversion of chitin to the tosylate. The reduction of these peaks for the azide-chitin intermediate, together with the appearance of a strong, sharp peak at 2110 cm^−1^ (azide stretch), signifies conversion of the tosyl group to the azide. For the amine- and alcohol-chitin derivatives, the 2110 cm^−1^ azide band is strongly reduced but remains detectable as a weak residual band, indicating that most, but not all, of the azide groups were converted to the corresponding triazole derivative via the CuAAC “click” reaction.

In the ^13^C NMR spectrum of unmodified chitin, the signals at 24, 56, 62, 74, 77, 84, 105, and 174 ppm correspond to the acetyl CH_3_, C2, C6, C3, C5, C4, C1, and C=O moieties, respectively [6]. For the amine-chitin derivative, broadening and a relative intensity increase of the C6 peak are associated with the addition of the triazole-tethered amine ligand, and a slight peak at 132 ppm indicates the C=C signal of the triazole tether [52]. The ^13^C NMR spectrum of alcohol-chitin is similar: the C6 peak is broadened with greater relative intensity, and the triazole C=C peak appears at 133 ppm. In addition, the C6 peak is shifted, as is the C=O peak of the amide acetyl group, which moves from 174 to 179 ppm. The triazole-tethered hydroxyl functionality may reestablish a small degree of new hydrogen bonding, influencing the chemical environment to a greater extent; this would also help explain the slight increase in thermal stability of alcohol-chitin over amine-chitin observed by TGA. The observed peak shifts for the derivatives relative to unmodified chitin agree with the C-6 functionalization of chitin performed by Zhang et al. [29].

**Figure 7:**
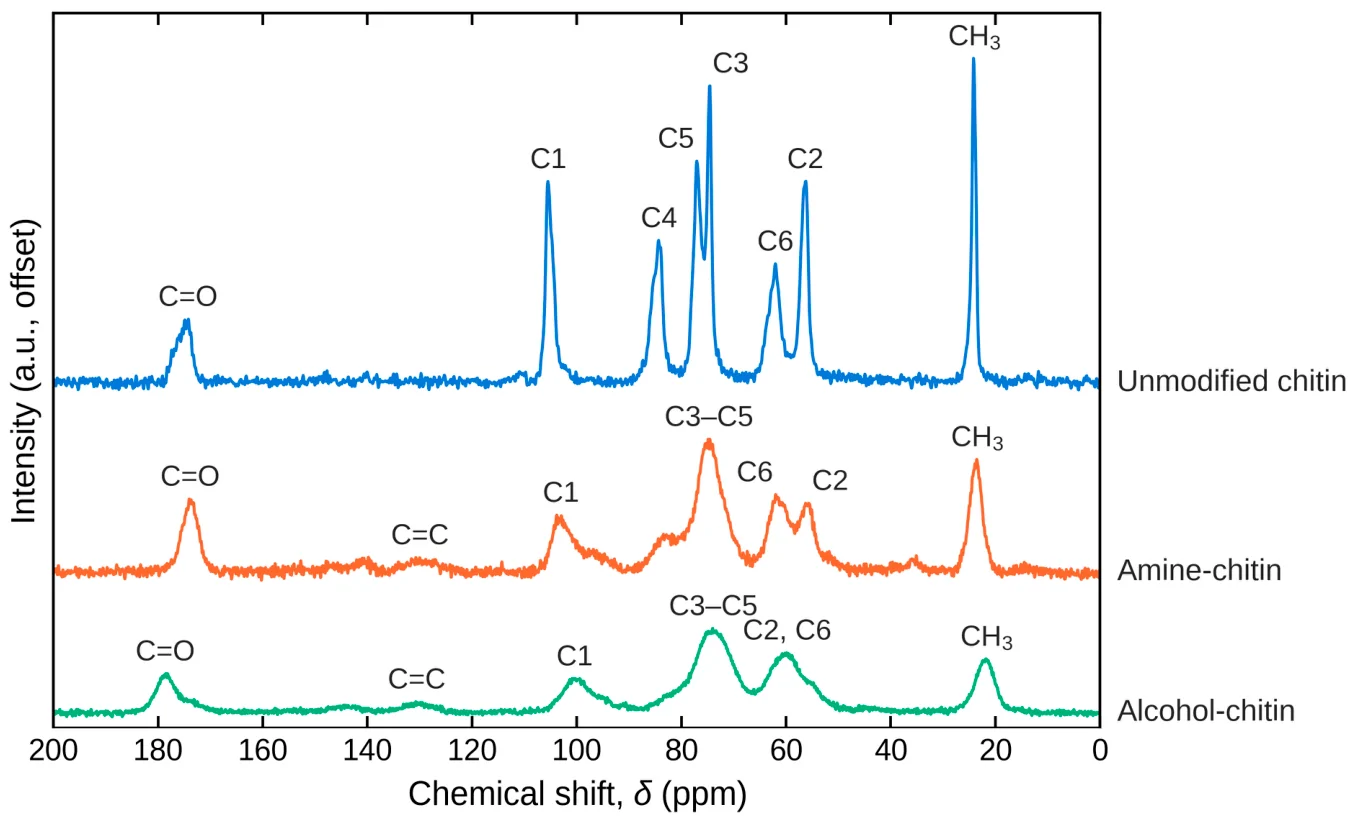
Solid-state ^13^C nuclear magnetic resonance (NMR) spectra of unmodified chitin (top), amine-chitin (middle), and alcohol-chitin (bottom), offset vertically for clarity, with carbon assignments labeled; “C=C” marks the triazole ring carbons. Spectra were recorded on a Bruker DSX-500 at 8 kHz with a 1.0 ms contact time.

Both adsorbents were prepared from a single batch of azide-chitin. In both, the azide stretching band at 2110 cm^−1^ was strongly reduced after the click reaction, although a weak residual band remained (Figure 6), and signals attributable to the triazole ring carbons appeared in the ^13^C NMR spectra (Figure 7). Conversion of the azide groups was therefore extensive but incomplete. Provided the azide groups were consumed by cycloaddition rather than by side reactions, the ligand loading of each adsorbent is somewhat below the azide content of the common intermediate, and because the residual band is weak in both, the two adsorbents are expected to carry similar, though not necessarily identical, ligand loadings. The absolute degree of substitution was not determined; the comparison between the two adsorbents is accordingly one of ligand identity at similar loading.

Because the derivatives were prepared with a copper catalyst, residual catalyst copper is a potential source of error in a Cu(II) adsorption study. After the acid wash, each adsorbent was soaked twice in water at pH 7 for 4 h, and the second soak solution contained no copper above the ICP-OES detection limit (0.004 mg L^−1^), showing that any catalyst copper remaining in the solids does not leach measurably under near-neutral conditions. Moreover, residual catalyst copper cannot inflate the reported uptakes: it is present in the solid before contact with the Cu(II) solution and does not deplete that solution, and any release during adsorption would raise the measured equilibrium concentration and so lower, rather than raise, the calculated uptake. The reported capacities are therefore conservative with respect to residual catalyst. The copper content of the solids themselves was not determined.

### 3.2. Effect of Adsorbent Dose on Cu(II) Adsorption

**Figure 8:**
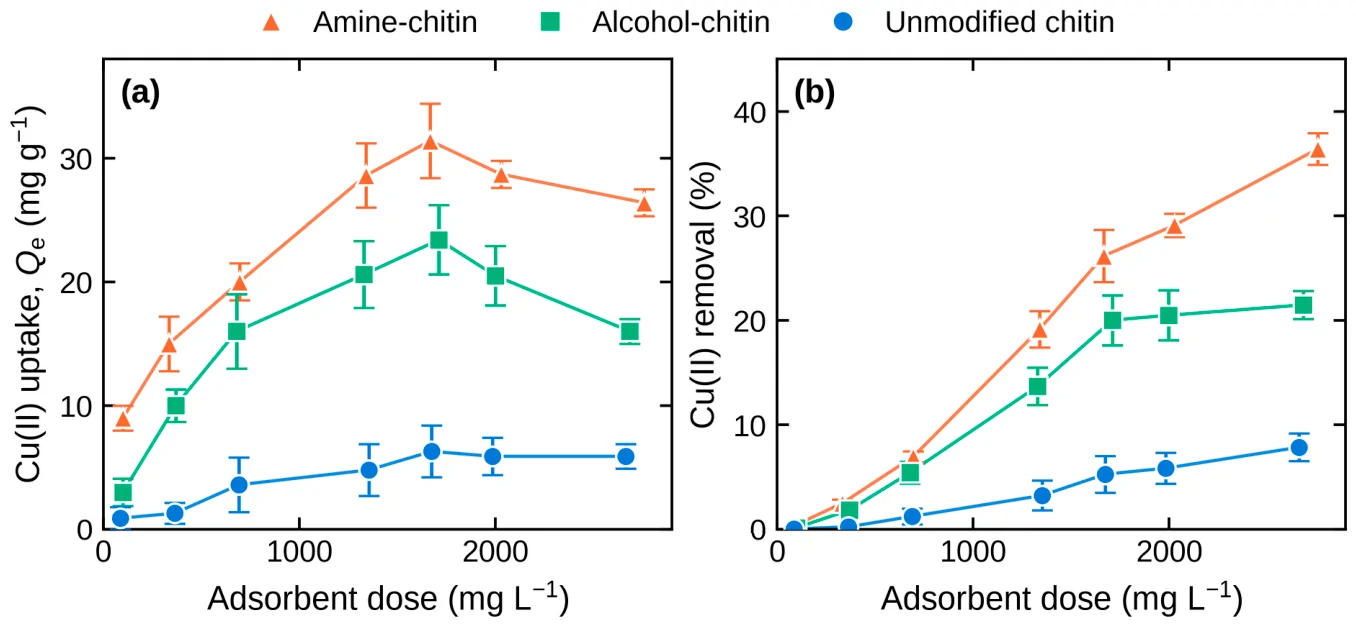
Effect of adsorbent dose on Cu(II) adsorption by amine-chitin, alcohol-chitin, and unmodified chitin: (**a**) uptake per unit mass (*Q*_e_) and (**b**) percentage of Cu(II) removed from solution. Conditions: 200 mg L^−1^ Cu(II), 15.0 mL, pH 6.0, 25 °C, 4 h contact. Points are means ± standard deviation (SD, *n* = 3); lines are guides to the eye.

The effect of adsorbent dose over 100–2700 mg L^−1^ at 200 mg L^−1^ Cu(II), pH 6.0, and 25 °C is shown in Figure 8, both as uptake per unit mass (Figure 8a) and as the percentage of Cu(II) removed from solution (Figure 8b). At every dose, uptake followed the order amine-chitin > alcohol-chitin > unmodified chitin. Uptake per unit mass was highest at approximately 1700 mg L^−1^ for all three materials—31.4 ± 3.0, 23.4 ± 2.8, and 6.3 ± 2.1 mg g^−1^ for amine-chitin, alcohol-chitin, and unmodified chitin, respectively—and decreased at higher doses, significantly for alcohol-chitin (16.0 ± 1.0 mg g^−1^ at 2685 mg L^−1^; *p* = 0.03) but not for amine-chitin (26.4 ± 1.1 mg g^−1^ at 2759 mg L^−1^; *p* = 0.09) or unmodified chitin. This decrease is expected, since at higher doses more Cu(II) is removed and the adsorbent is exposed to a lower equilibrium concentration. The percentage of Cu(II) removed increased with dose, reaching 36% for amine-chitin at 2760 mg L^−1^, whereas for alcohol-chitin it leveled off at 20–21% above 1700 mg L^−1^, and for unmodified chitin it did not exceed 8%.

At doses below approximately 1300 mg L^−1^, uptake per unit mass was markedly lower than at 1700 mg L^−1^ (*p* ≤ 0.03 for all three materials). This is contrary to the behavior expected at a fixed initial concentration: the lowest doses deplete the solution least, so the adsorbent is exposed to the highest equilibrium concentration, and the isotherm (Section 3.4) predicts uptakes of approximately 32 and 24 mg g^−1^ for amine- and alcohol-chitin under these conditions, against 9 and 3 mg g^−1^ measured at 100 mg L^−1^. The origin of this discrepancy was not established. At 100 mg L^−1^ the uptake is derived from a concentration change of less than 1 mg L^−1^ in a 200 mg L^−1^ solution, and incomplete dispersion of the small adsorbent masses (1.5–5.5 mg) may also contribute; the low-dose results should therefore be interpreted with caution.

The dose of approximately 1700 mg L^−1^, at which uptake per unit mass was greatest, was adopted as the standard dose for characterizing and comparing the adsorbents. This criterion maximizes utilization of the adsorbent rather than removal from solution; the dose required in an application would depend on the treatment objective, with greater removal obtained at higher doses at the cost of lower uptake per unit mass.

### 3.3. Effect of pH on Cu(II) Adsorption

**Figure 9:**
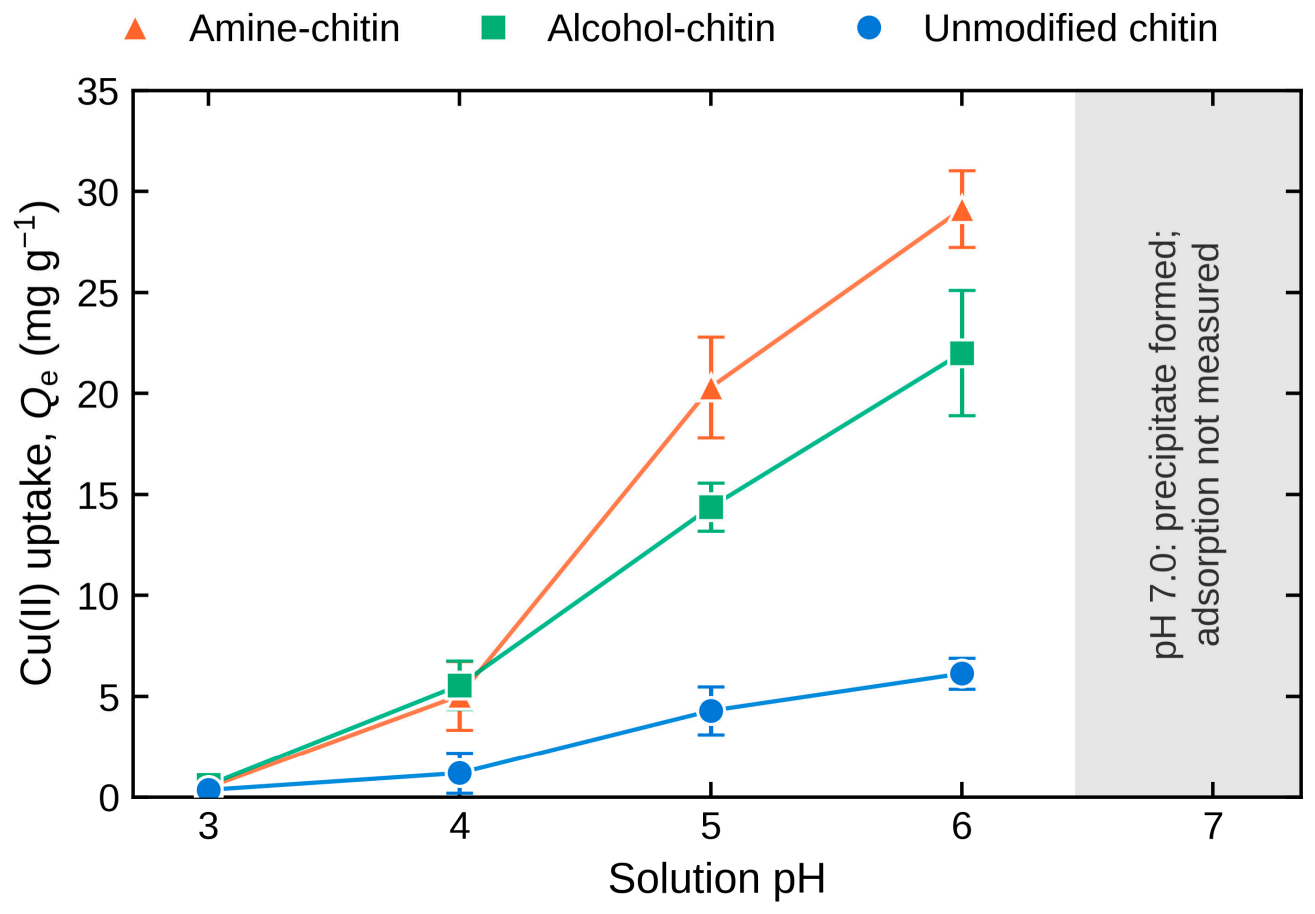
Effect of solution pH on Cu(II) uptake (*Q*_e_) by amine-chitin, alcohol-chitin, and unmodified chitin. Conditions: 25.0 mg of adsorbent in 15.0 mL of 200 mg L^−1^ Cu(II), 25 °C, 4 h contact. Points are means ± standard deviation (SD, *n* = 3); lines are guides to the eye. At pH 7.0 (shaded), a precipitate formed in the adsorbent-free controls and adsorption was not measured.

Cu(II) uptake over pH 3.0–6.0 is shown in Figure 9. For all three materials uptake was negligible at pH 3.0 and increased with pH to 6.0, reaching 29.1, 22.0, and 6.1 mg g^−1^ for amine-chitin, alcohol-chitin, and unmodified chitin, respectively. The increase coincides with two changes that the present data cannot separate: decreasing competition from H_3_O^+^ for the ligand donor atoms as pH rises, and the shift of the adsorbent surface charge to negative values below pH 4 for both derivatives (Section 3.1, Figure 5). At pH 6.0 the primary amine of the amine-chitin ligand is expected to be largely protonated, which argues against electrostatic attraction of Cu^2+^ as the dominant interaction and is more consistent with coordination by the triazole nitrogen and ligand donor atoms; the binding mode was not determined in this work.

Cu(II) speciation under the experimental conditions was calculated from the recommended hydrolysis and solubility constants of Powell et al. [53], corrected to the ionic strength of the experimental solutions (~0.01 M) with the Davies equation; complexation by nitrate was neglected (Figure S1). At 200 mg L^−1^, dissolved Cu(II) is present almost entirely as the free aquo ion up to pH 5.5, and as approximately 87% free Cu^2+^ at pH 6.0, where the dinuclear hydroxo complex Cu_2_(OH)_2_^2+^ accounts for most of the remainder. The solutions reach saturation with respect to Cu(OH)_2_(s) at pH ≈ 5.7, are modestly supersaturated at pH 6.0 (saturation index ≈ +0.6), and are strongly supersaturated at pH 7.0 (≈+1.9). Because freshly precipitated, poorly crystalline copper hydroxides are more soluble than the crystalline reference phase [53], these indices are upper bounds on the driving force for precipitation, consistent with solutions at pH 6.0 remaining largely metastable over the 4 h contact period.

The adsorbent-free controls are consistent with this calculation. At pH 6.0, dissolved Cu(II) in the controls decreased by approximately 3% over 4 h; at pH 7.0 a precipitate formed and accumulated visibly at the bottom of the control vessels. Because precipitated Cu(II) would be removed with the adsorbent during separation and register as depletion of the solution [54], uptake at pH 7.0 could not be attributed to adsorption, and adsorption was not measured at this pH. pH 6.0, the highest pH at which no precipitate was observed, was adopted as the working pH.

The ~3% loss at pH 6.0 corresponds to up to 3.6 mg g^−1^ of apparent uptake at the standard adsorbent dose. This is an upper bound on the contribution of precipitation to the uptakes reported at this pH, since the dissolved Cu(II) concentration, and hence the degree of supersaturation, is lower in the adsorbent vessels than in the controls. It is small relative to the uptakes of the derivatives (at most 12% and 16%) but comparable to that of unmodified chitin (up to 58%). Uptakes are reported without correction; the fold-increases of the derivatives over unmodified chitin (Section 3.5) are accordingly conservative, since any correction would reduce the chitin uptake proportionally more than those of the derivatives.

Taken together, the characterization data constrain the origin of the difference between the two derivatives. Both were prepared from the same azide-chitin and are therefore expected to carry similar ligand loadings (Section 3.1); both are essentially X-ray amorphous (crystallinity index 8% and 4%); their surface morphologies are similar (Figure 3); and their isoelectric points are similar (pH ≈ 3.8 and 3.5), with both surfaces carrying a small negative charge at pH 6.0 (approximately −2.1 and −1.4 mV, respectively; Figure 5). The triazole ring is common to both. The approximately 35% higher uptake of amine-chitin, using the mean across experiments as the reference point (30.1 versus 22.3 mg g^−1^), is therefore most plausibly associated with the terminal donor group, although the binding mode was not determined and a difference in the accessibility of the two ligands cannot be excluded. The larger difference between both derivatives and unmodified chitin cannot, by contrast, be assigned to a single factor: introduction of the triazole and terminal donor groups, the loss of crystalline order, which may increase the accessibility of the polymer chains, and the shift of the isoelectric point to lower pH all change together on functionalization, and the present data do not separate their contributions.

### 3.4. Effect of Solution Concentration on Adsorption

**Figure 10:**
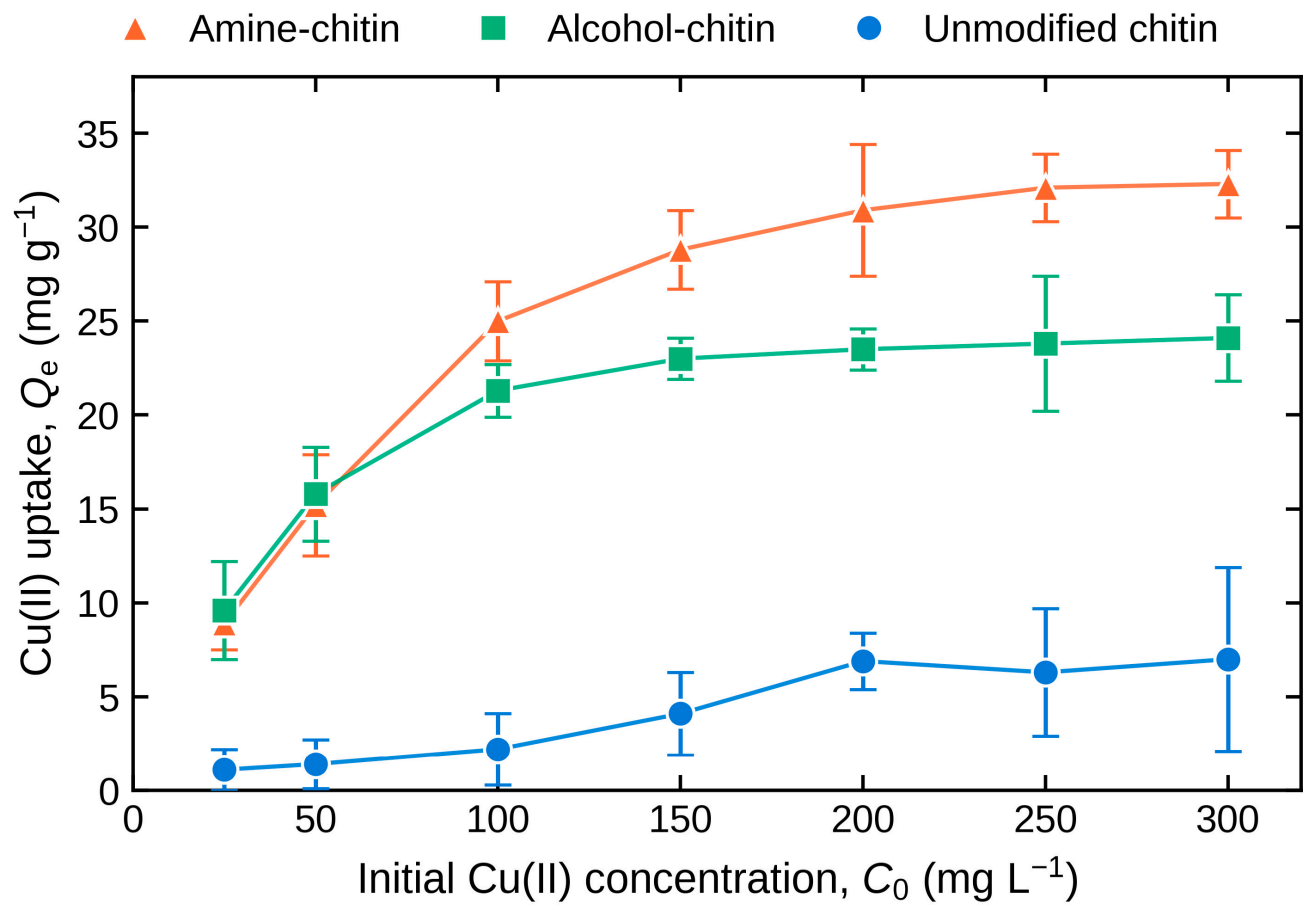
Cu(II) uptake (*Q*_e_) by amine-chitin, alcohol-chitin, and unmodified chitin as a function of initial Cu(II) concentration (*C*_0_). Conditions: 25.0 mg of adsorbent in 15.0 mL, pH 6.0, 25 °C, 4 h contact. Points are means ± standard deviation (SD, *n* = 3); lines are guides to the eye.

Adsorption isotherms of amine-chitin and alcohol-chitin were collected over initial Cu(II) concentrations of 25–300 mg L^−1^ at pH 6.0 and 25 °C, using 25.0 mg of adsorbent in 15.0 mL of solution and the 4 h contact time adopted in Section 2.4 (Figure 10). Uptake increased with concentration for both derivatives and began to level off above approximately 200 mg L^−1^, beyond which further increases in concentration produced only small increases in uptake, consistent with progressive occupation of the available adsorption sites. The highest uptakes measured in the isotherm series, at C_0_ = 300 mg L^−1^, were 32.3 and 24.1 mg g^−1^ for amine- and alcohol-chitin, respectively.

**Figure 11:**
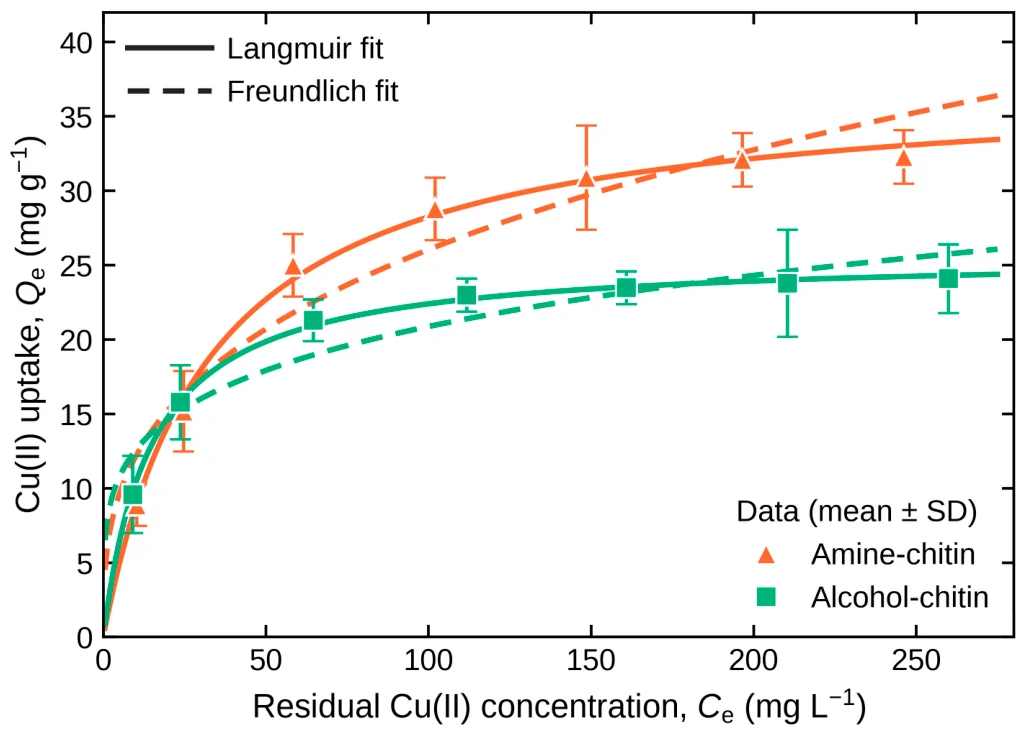
Cu(II) uptake (*Q*_e_) by amine-chitin and alcohol-chitin as a function of the residual Cu(II) concentration after 4 h contact (*C*_e_), with nonlinear least-squares fits of the Langmuir (solid lines) and Freundlich (dashed lines) models; fitted parameters are given in Table 2. Points are means ± standard deviation (SD, *n* = 3) of *Q*_e_; *C*_e_ is calculated from the same measurements (Equation (1)), so its uncertainty is not shown separately.

The isotherm data were fitted to the Freundlich (Equation (4)) and Langmuir (Equation (5)) models by nonlinear least-squares regression of Q_e_ against C_e_:

(4) $$\mathrm{Freundlich}:\;Q_{e}\;=\;K_{F}C_{e}^{\frac{1}{n}}$$

(5) $$\mathrm{Langmuir}:\;Q_{e}=\frac{bQ_{\max}C_{e}}{1+bC_{e}}$$

where Q_max_ is the Langmuir capacity parameter (mg g^−1^), b is the Langmuir affinity constant (L mg^−1^), K_F_ is the Freundlich constant, and n is the Freundlich intensity parameter. Because uptake was measured after 4 h of contact rather than at a demonstrated equilibrium (Section 2.4), the fitted parameters describe the 4 h contact-time isotherm and should be regarded as apparent values. Parameters with standard errors, the coefficient of determination, and the corrected Akaike information criterion (AICc) are given in Table 2, and the fitted curves in Figure 11. Fits weighted by the replicate standard deviations gave the same parameters within uncertainty and supported the same model choice (reduced χ^2^ of 0.11 and 0.02 for the Langmuir model, against 2.3 and 1.0 for the Freundlich model; Table S2).

For both derivatives the Langmuir model describes the data substantially better than the Freundlich model (amine-chitin: R^2^ = 0.995 versus 0.923, ΔAICc = 18.9; alcohol-chitin: R^2^ = 0.998 versus 0.894, ΔAICc = 28.0). The Langmuir capacity parameters, 37.4 ± 0.8 and 25.7 ± 0.2 mg g^−1^, exceed the highest uptakes measured by 16% and 7%, respectively, indicating that neither isotherm had fully reached saturation at 300 mg L^−1^. The Freundlich intensity parameters (n = 3.0 ± 0.5 and 4.6 ± 0.8) and the Langmuir separation factors, R_L_ = 1/(1 + bC_0_), of 0.10–0.56 and 0.05–0.37 over the concentration range both fall within the range conventionally associated with favorable adsorption.

Agreement with the Langmuir equation is consistent with saturable uptake at a finite number of sites, as would be expected for adsorption to discrete grafted ligands. It does not by itself establish that the adsorbent surface is energetically homogeneous or that adsorption is confined to a monolayer, and no such inference is drawn here [55].

Table 3 compares the present adsorbents with chitin-, chitosan-, and other biopolymer-based Cu(II) adsorbents reported previously. Such comparisons are approximate, because capacities were determined under different pH, contact time, dose, concentration range, and temperature, and some are Langmuir capacities while others are uptakes at a single concentration [56]. With that caveat, the capacities of amine- and alcohol-chitin lie within the range reported for chitin-based adsorbents but below those of chemically modified chitin, expanded chitin foams, and chitosan beads, and well below that of the amidoxime–triazole cotton prepared by an analogous CuAAC route [38]. Reported capacities for unmodified chitin (13.8–58 mg g^−1^) are also higher than the 6.2 mg g^−1^ measured here, which may reflect differences in chitin source, degree of acetylation (>98% here), particle form, and experimental conditions. The value of the present materials therefore lies not in absolute capacity but in the insolubility of the highly acetylated backbone across the working pH range and in the modularity of the click route, which allows the ligand to be exchanged on a common backbone.

### 3.5. Effect of Contact Time and Adsorption Kinetics

**Figure 12:**
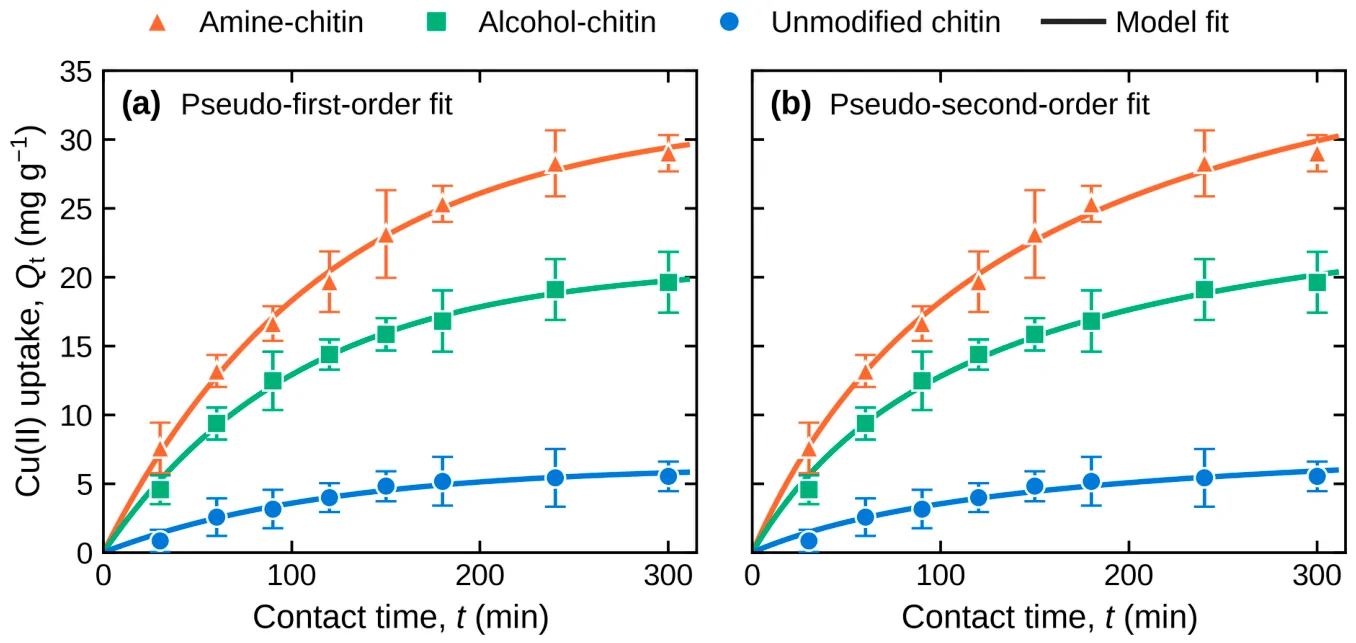
Cu(II) uptake (*Q*_t_) as a function of contact time (*t*) for amine-chitin, alcohol-chitin, and unmodified chitin, with nonlinear least-squares fits (solid lines) of (**a**) the pseudo-first-order and (**b**) the pseudo-second-order models. The data are the same in both panels; fitted parameters are given in Table 4. Conditions: 85.0 mg of adsorbent in 50.0 mL of 200 mg L^−1^ Cu(II) (1700 mg L^−1^ adsorbent), pH 6.0, 25 °C. Points are means ± standard deviation (SD, *n* = 3 independent vessels per contact time).

Uptake-versus-time data for unmodified chitin, amine-chitin, and alcohol-chitin were collected at 30, 60, 90, 120, 150, 180, 240, and 300 min under the standard conditions described in Section 2.4 (200 mg L^−1^ Cu(II), 1700 mg L^−1^ adsorbent, pH 6.0, 25 °C), each point being the mean of three independent vessels (Figure 12). Uptake rose steeply over the first 2 h and leveled off from approximately 4 h, reaching 28.3 ± 2.4 mg g^−1^ for amine-chitin, 19.1 ± 2.2 mg g^−1^ for alcohol-chitin, and 5.4 ± 2.1 mg g^−1^ for unmodified chitin at 4 h, with only minor further change by 5 h. The concave uptake profile is consistent with a progressively decreasing number of available adsorption sites and a declining concentration driving force as adsorption proceeds [60]. Contact beyond 5 h was examined separately at 24 and 36 h; as described in Section 2.4, no extended-contact value differed significantly from the corresponding 4 h value, and 4 h was adopted as the standard operational contact time.

The contact-time data were fitted to the pseudo-first-order (Equation (6)) and pseudo-second-order (Equation (7)) [61] rate expressions:

(6) $$Q_{t}\;=\;Q_{e}\left(1\;-\;e^{-k_{1}t}\right)$$

(7) $$Q_{t}=\frac{k_{2}{Q_{e}}^{2}t}{1+k_{2}Q_{e}t}$$

where Q_e_ is the fitted limiting uptake (mg g^−1^), Q_t_ the uptake at time t, and k_1_ (min^−1^) and k_2_ (g mg^−1^ min^−1^) the rate constants. Both expressions were fitted directly to the untransformed uptake data by nonlinear least-squares regression rather than through their linearized forms, because the linearizing transformations redistribute the residuals and are known to bias the resulting fit statistics in favor of the pseudo-second-order model [60,62]. Fitted parameters, with standard errors and the corrected Akaike information criterion (AICc), are given in Table 4, and the fitted curves are shown with the data in Figure 12 (Figure 12a, pseudo-first-order; Figure 12b, pseudo-second-order).

For all three materials the pseudo-first-order expression is the better-supported description of the data, with the lower AICc in each case (ΔAICc = 3.0 for amine-chitin, 5.7 for alcohol-chitin, and 2.9 for unmodified chitin), although the difference in coefficient of determination between the two models is small (R^2^ = 0.996 versus 0.994 for amine-chitin, for example). The distinction is clearer in the fitted capacities: the pseudo-first-order Q_e_ values of 31.9 ± 0.7 and 20.9 ± 0.5 mg g^−1^ for amine- and alcohol-chitin are consistent with the measured uptake at the plateau, whereas the corresponding pseudo-second-order values of 44.0 ± 1.8 and 28.3 ± 1.3 mg g^−1^ exceed the measured uptake at 5 h by 52 and 44%, respectively. The same ranking is obtained when the fits are weighted by the replicate standard deviations.

Two limitations of this analysis should be noted. First, both expressions are empirical rate laws, and agreement with either is observed for a very wide range of adsorbate–adsorbent systems; the quality of fit does not by itself identify the rate-limiting step or the nature of the adsorbate–adsorbent interaction, and no mechanistic inference is drawn from it here. Adsorption onto a porous or swollen solid generally involves external mass transfer, diffusion within the particle, and binding at the surface, and these steps may proceed simultaneously; the present data, collected at a single initial concentration and particle size, do not allow their contributions to be separated. The binding mode was not determined spectroscopically in this work and remains to be established. Second, the fitted parameters describe the 30–300 min interval over which the data were collected and should not be extrapolated beyond it: the pseudo-first-order Q_e_ for alcohol-chitin, for example, lies below the uptake subsequently measured at 24 and 36 h.

Uptake at 4 h in the kinetic series, which used larger vessels (50 mL), was consistent within uncertainty with that measured at the same nominal conditions in the pH, dose, isotherm, and regeneration experiments (*p* ≥ 0.06; Table S1). Comparisons between adsorbents are based on the mean across these experiments and on uptake ratios, which are consistent across all of them (4.5–5.2 for amine-chitin and 3.4–3.7 for alcohol-chitin relative to unmodified chitin).

### 3.6. Adsorbent Regeneration

**Figure 13:**
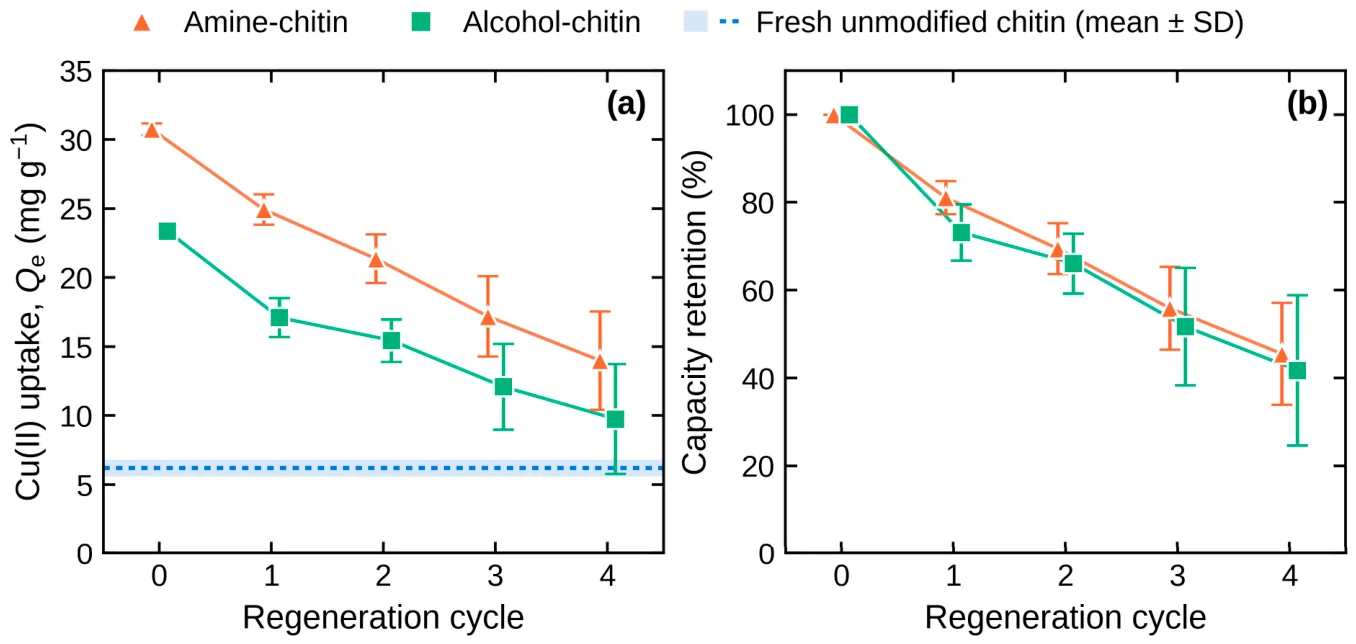
Regeneration of amine-chitin and alcohol-chitin with 7% (*v*/*v*) HCl: (**a**) Cu(II) uptake (*Q*_e_) in the initial adsorption (cycle 0, fresh adsorbent) and after each of four regenerations, and (**b**) capacity retention relative to cycle 0. The dotted line and shaded band in (**a**) show the uptake of fresh unmodified chitin under the same conditions (mean ± SD across experiments; Table S1). Points are means ± standard deviation (SD, *n* = 3); retention uncertainties are propagated from the SDs; symbols are offset horizontally for clarity.

The reusability of the derivatives was evaluated over an initial adsorption and four subsequent adsorption–desorption cycles (Figure 13, Table S6). Uptake declined with each cycle for both derivatives. Amine-chitin adsorbed 30.8 ± 0.4 mg g^−1^ in the initial cycle and 24.9 ± 1.1, 21.4 ± 1.8, 17.2 ± 2.9, and 14.0 ± 3.6 mg g^−1^ in the four subsequent cycles, retaining 46 ± 12% of its initial capacity; alcohol-chitin adsorbed 23.4 ± 0.7 mg g^−1^ initially and 17.1 ± 1.4, 15.5 ± 1.5, 12.1 ± 3.1, and 9.8 ± 4.0 mg g^−1^ thereafter, retaining 42 ± 17%. The decline over four cycles was significant for both materials (*p* = 0.014 and 0.025, respectively; Welch’s *t*-test) and approximately constant in proportion from cycle to cycle, averaging 18% per cycle for amine-chitin and 20% for alcohol-chitin. Variability among replicates increased markedly with cycling, from relative standard deviations of 1–3% in the initial cycle to 25–41% after the fourth regeneration. After four regenerations, the uptake of neither derivative differed significantly from that of fresh unmodified chitin under the same conditions.

The cause of the decline was not determined. Because uptake was normalized to the recovered adsorbent mass in each cycle, physical loss of adsorbent during recovery does not account for it; the decline may instead reflect incomplete desorption of Cu(II) by the acid wash, loss or degradation of the grafted ligand under repeated acid exposure, or both. Desorption efficiency was not measured, and neither the Cu(II) content of the eluates nor the structure of the cycled adsorbents was analyzed, so these contributions cannot be distinguished. The increasing replicate variability suggests that whatever process is responsible proceeds at different rates in nominally identical samples. The results show that the derivatives can be regenerated and reused with partial retention of capacity, but not that they are stable to repeated regeneration under these conditions, and optimization of the regeneration protocol, including eluent, concentration, and contact time, remains to be carried out.

### 3.7. Thermal Stability

**Figure 14:**
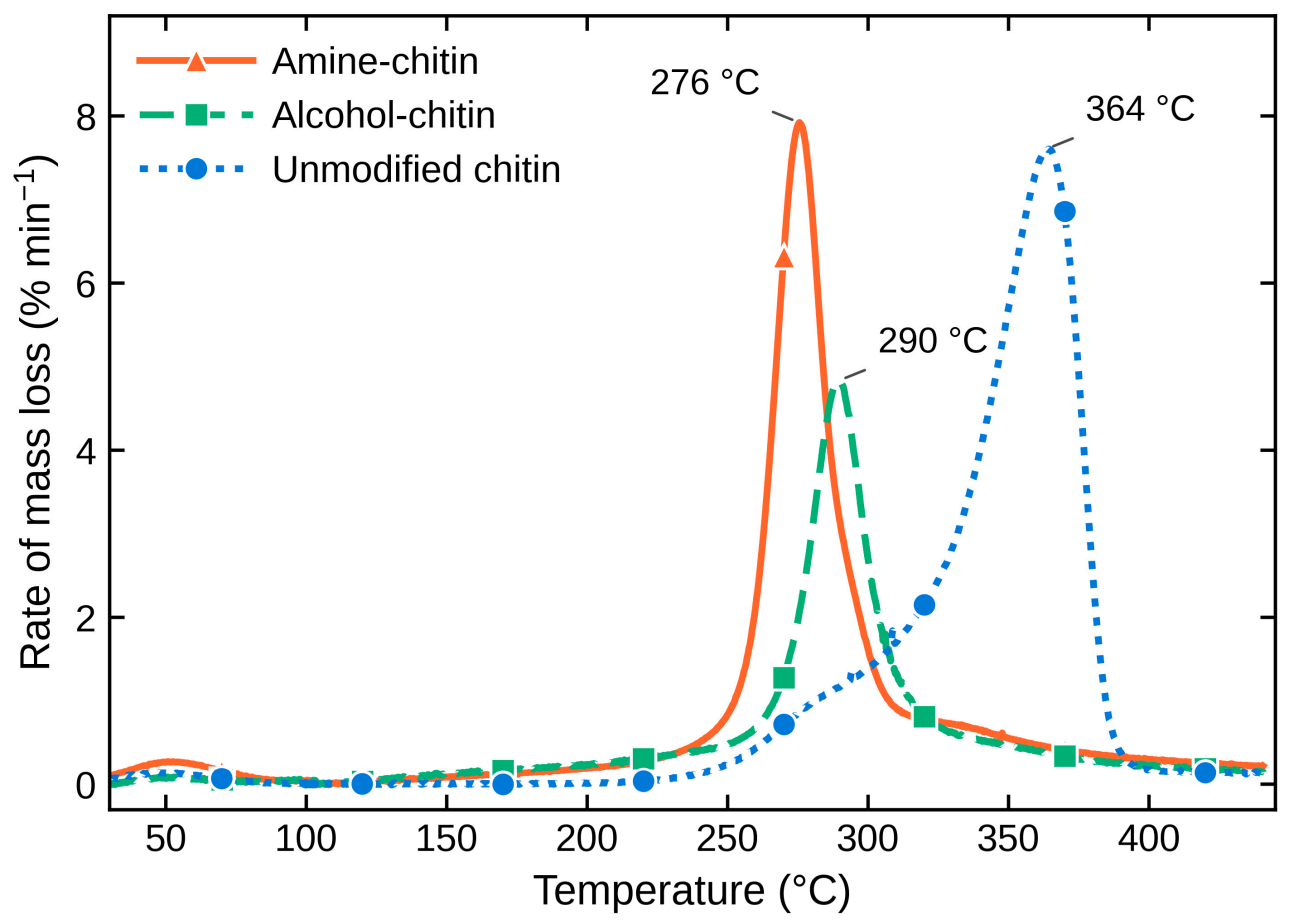
Derivative thermogravimetric (DTG) curves (rate of mass loss) of amine-chitin, alcohol-chitin, and unmodified chitin with a ramp rate of 5 °C min^−1^. The temperatures of maximum rate are marked: 276, 290, and 364 °C, respectively.

**Figure 15:**
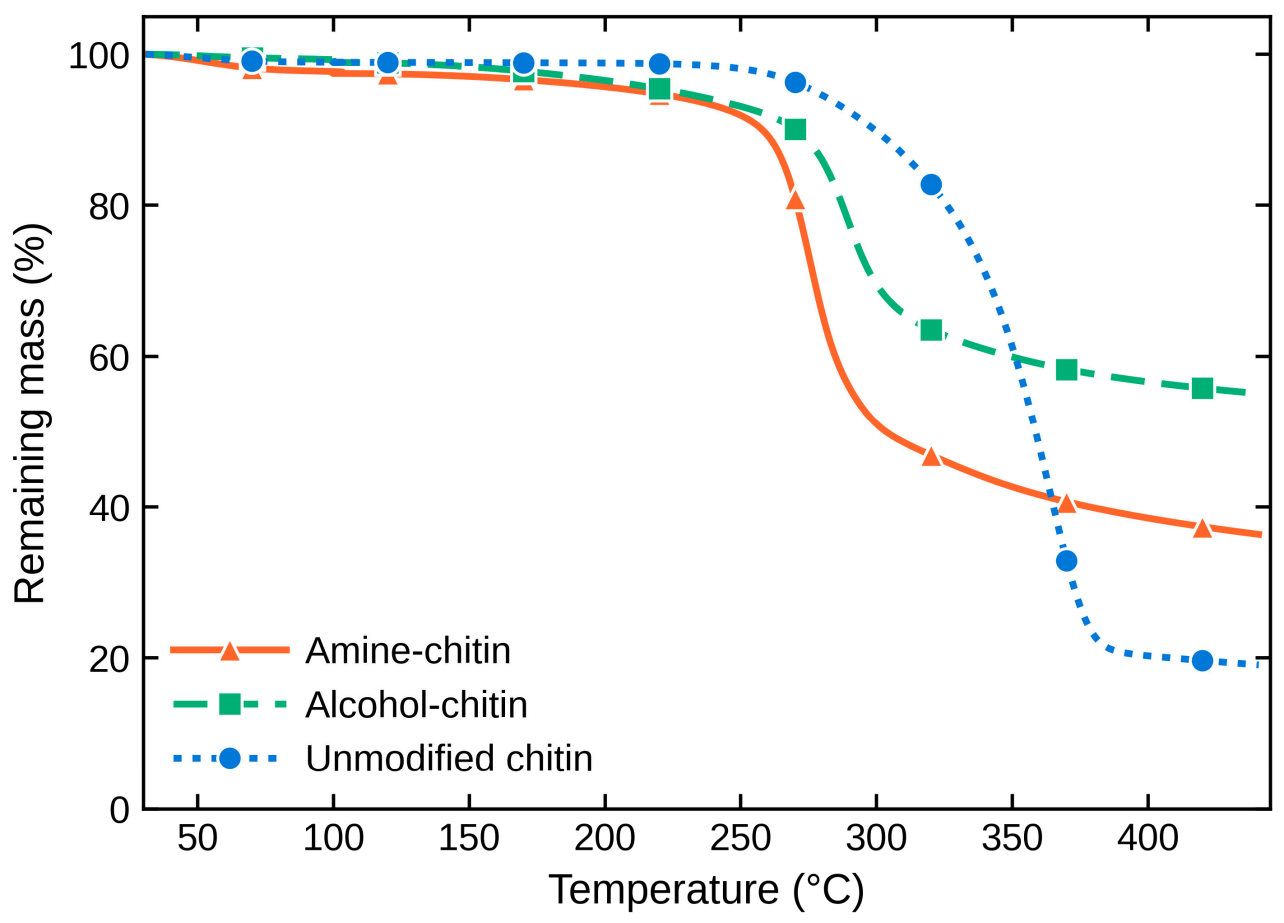
Thermogravimetric analysis (TGA) curves (remaining mass versus temperature) of amine-chitin, alcohol-chitin, and unmodified chitin at a ramp rate of 5 °C min^−1^.

Thermal decomposition profiles are shown in Figure 14 and Figure 15 (5 °C min^−1^). After loss of adsorbed water below 150 °C (1–3% of the initial mass), decomposition of both derivatives began at lower temperatures than that of unmodified chitin: on a dry-mass basis, 5% mass loss occurred at 248 and 246 °C for amine- and alcohol-chitin, respectively, against 283 °C for unmodified chitin. The temperature of maximum mass-loss rate was likewise lower for amine-chitin (276 °C) and alcohol-chitin (290 °C) than for unmodified chitin (364 °C) (Figure 14). The main decomposition step was largely complete by approximately 320 °C for amine-chitin and 350 °C for alcohol-chitin, with mass losses at 400 °C of 61% and 43%, respectively, compared with about 80% for unmodified chitin, whose decomposition was complete near 385 °C (Figure 15). The reduced thermal stability of the derivatives is consistent with the loss of crystalline order described in Section 3.1, which removes much of the intermolecular and intramolecular hydrogen bonding that stabilizes α-chitin [29]. The greater residual mass of the derivatives may reflect a greater tendency of the substituted material to form char; the composition of the residue was not determined, and a contribution from residual inorganic material cannot be excluded.

## 4. Conclusions

Two chitin-based adsorbents bearing triazole-tethered amine and hydroxyl ligands were prepared from highly acetylated chitin by tosylation, azidation, and CuAAC coupling. The route comprises three steps without chromatographic purification, and because both products derive from a common azide intermediate, ligands can be compared on a common backbone. Functionalization disrupted the crystalline structure of α-chitin, principally at the tosylation step, and the click products were essentially X-ray amorphous, with correspondingly reduced thermal stability.

Under standard batch conditions of 1700 mg L^−1^ adsorbent dose, 200 mg L^−1^ Cu(II), pH 6.0, and a 4 h contact time, amine-chitin and alcohol-chitin adsorbed 30.1 ± 1.3 and 22.3 ± 1.9 mg Cu g^−1^, respectively, representing 4.9 ± 0.3- and 3.6 ± 0.1-fold increases over unmodified chitin, ratios that were consistent across independent adsorption experiments. pH 6.0 was the highest pH at which no Cu(II) precipitate was observed; above it, precipitation precludes meaningful adsorption measurements. Isotherm data at 4 h contact were well described by the Langmuir equation, with apparent capacities of 37.4 ± 0.8 and 25.7 ± 0.2 mg g^−1^, and contact-time data were better described by the pseudo-first-order than the pseudo-second-order equation. These empirical fits are consistent with saturable uptake at a finite number of sites but do not identify the binding mode, which was not determined. The adsorbents could be regenerated with 7% (*v*/*v*) HCl and reused, but lost roughly half of their capacity over four cycles, retaining 46 ± 12% and 42 ± 17% of their initial uptake.

Several aspects remain to be established before these materials can be assessed for practical water treatment: the absolute ligand loading and the Cu(II) binding mode; uptake at equilibrium beyond the 4 h operational contact time; the origin of the capacity loss on regeneration and conditions that reduce it; and performance in the presence of competing ions and in real wastewaters. The synthesis also carries a substantial reagent burden, including the DMAc/LiCl solvent system, excess tosyl chloride, sodium azide, and a copper catalyst, so solvent recovery, reduced reagent excess, and catalyst minimization would be prerequisites for any scale-up. Within these limits, the work shows that the click route provides a practical means of attaching interchangeable functional groups to highly acetylated chitin without deacetylation, and that the adsorption behavior of the resulting materials can be varied through the choice of ligand, offering a route toward application-specific adsorbents from an underutilized biopolymer.

## Figures and Tables

**Table 1:** Crystallinity index and apparent crystallite size normal to the (110) plane ^1^.

Material	Crystallinity Index (%)	Crystallite Size (nm)
Chitin	83	6.9
Tosyl-chitin	25	2.5
Azide-chitin	42	2.6
Amine-chitin	8	n.d.
Alcohol-chitin	4	n.d.

^1^ Scherrer equation with K = 0.9 and λ = 0.179 nm (Co Kα); no correction for instrumental broadening. Standard errors from the fit are ±0.1, ±0.3, and ±0.6 nm; alternative fitting windows and profile models give 6.9–8.8, 1.6–3.0, and 2.2–2.7 nm, respectively, without changing the trend. n.d.: (110) reflection not resolved with sufficient signal-to-noise.

**Table 2:** Freundlich and Langmuir parameters for Cu(II) adsorption on amine-chitin and alcohol-chitin ^1^.

Adsorbent	K_F_	n	R^2^	AICc	Q_max_ (mg g^−1^)	b (L mg^−1^)	R^2^	AICc
Amine-chitin	5.7 ± 1.5	3.0 ± 0.5	0.923	19.0	37.4 ± 0.8	0.0309 ± 0.0024	0.995	0.1
Alcohol-chitin	7.6 ± 1.5	4.6 ± 0.8	0.894	14.0	25.7 ± 0.2	0.0683 ± 0.0026	0.998	−14.0

^1^ Columns 2–5: Freundlich model; columns 6–9: Langmuir model. Estimate ± standard error from unweighted nonlinear least-squares regression of Q_e_ on C_e_ (seven initial concentrations, 25–300 mg L^−1^; 25.0 mg adsorbent in 15.0 mL; 4 h contact). 95% confidence intervals are given in Table S2. The lower AICc indicates the better-supported model. Units: *K*_F_, (mg g^−1^)(L mg^−1^)^1/*n*^; *n*, dimensionless. AICc, corrected Akaike information criterion.

**Table 3:** Cu(II) adsorption capacities of chitin-, chitosan-, and other biopolymer-based adsorbents ^1^.

Adsorbent	Cu(II) Capacity (mg g^−1^)	Ref.
Acid-washed shrimp shell	16	[25]
Chitin flakes	13.8 ± 2.9	[26]
Chitin (shrimp shell)	24	[25]
Chitin (lobster waste)	58 ^2^	[28]
Chitin/L-cysteine-modified chitin	57.9/86.1 ^2^	[57]
Expanded chitin foam, oven-dried/lyophilized	48.6 ± 1.9/73.1 ± 2.0	[26]
NaOH-treated shrimp shell	45.8 ^3^	[58]
Chitosan beads/chitosan–alginate beads	64.6/67.7	[59]
Amidoxime–triazole cotton (CuAAC)	122.4	[38]
Unmodified chitin (this work)	6.2 ± 0.6 ^4^	—
Alcohol-chitin (this work)	25.7 ^5^	—
Amine-chitin (this work)	37.4 ^5^	—

^1^ Capacities were determined under different conditions and are not strictly comparable; see the cited sources. ^2^ As tabulated in Anastopoulos et al. [56];. ^3^ Langmuir capacity at 20 °C (66.1 mg g^−1^ at 50 °C). ^4^ Uptake at 200 mg L^−1^ and 4 h contact; no isotherm was fitted. ^5^ Apparent Langmuir capacity at 4 h contact.

**Table 4:** Pseudo-first-order and pseudo-second-order kinetic parameters for Cu(II) adsorption, obtained by nonlinear least-squares regression of the uptake data in Figure 12 ^1^.

Adsorbent	Q_e,exp_	Q_e_ (PFO)	k_1_ (min^−1^)	R^2^	AICc	Q_e_ (PSO)	k_2_ (g mg^−1^ min^−1^)	R^2^	AICc
Amine-chitin	29.0	31.9 ± 0.7	(8.5 ± 0.4) × 10^−3^	0.996	−6.2	44.0 ± 1.8	(1.61 ± 0.21) × 10^−4^	0.994	−3.2
Alcohol-chitin	19.6	20.9 ± 0.5	(9.6 ± 0.5) × 10^−3^	0.995	−11.4	28.3 ± 1.3	(2.91 ± 0.45) × 10^−4^	0.990	−5.7
Chitin	5.5	6.3 ± 0.4	(8.4 ± 1.3) × 10^−3^	0.970	−14.6	8.9 ± 1.1	(7.35 ± 2.90) × 10^−4^	0.956	−11.7

^1^ Q_e_ in mg g^−1^; estimate ± standard error; Q_e,exp_ is the measured uptake at 300 min. Columns 3–6: pseudo-first-order (PFO) model; columns 7–10: pseudo-second-order (PSO) model. The lower AICc indicates the better-supported model. *k*_1_ and *k*_2_, pseudo-first-order and pseudo-second-order rate constants; AICc, corrected Akaike information criterion.

## Data Availability

The data supporting the conclusions of this article are included in the article and its Supplementary Materials. Raw instrument files (XRD, DRIFTS, solid-state NMR, TGA, and ICP-OES) are available from the corresponding author on reasonable request, subject to the terms of the sponsor agreement with the Army Research Laboratory.

## Supplementary Materials

The following supporting information can be downloaded at: https://www.mdpi.com/article/10.3390/polym18192450/s1, Table S1: Uptake at the standard condition across independent experiments; Table S2: 95% confidence intervals and weighted fits for the isotherm parameters; Table S3: Contact-time data with standard deviations; Table S4: pH-series uptakes and adsorbent-free control losses; Table S5: Dose-series uptakes and percentage removal; Table S6: Regeneration-cycle uptakes, standard deviations, and capacity retention; Figure S1: Calculated Cu(II) speciation and saturation index as a function of pH; Figure S2: Full X-ray diffraction patterns of chitin and all derivatives with the fitted (110) components.
