# Supplementary material for: Preparation of Customizable Ligand-Functionalized Chitin Biopolymer Adsorbents Utilizing the Azide–Alkyne “Click” Reaction for the Adsorption of Aqueous Cu(II)
Source: Polymers (Basel). 2026 Oct 8;18(19):2450. doi: 10.3390/polym18192450 (PMC13667407; doi:10.3390/polym18192450)
Supplement: Supplementary file 1 [file polymers-18-02450-s001.zip › polymers-4567572-supplementary.pdf]

Supplementary Materials

Preparation of Customizable Ligand-Functionalized Chitin Biopolymer Adsorbents Utilizing the Azide–Alkyne "Click" Reaction for the Adsorption of Aqueous Cu(II)

Blaine M. Berrington, Reyla Williams and Richard LaDouceur

Table S1. Cu(II) uptake (mg g^{-1}) at the standard condition (200 mg L^{-1} Cu(II), ~1700 mg L^{-1} adsorbent, pH 6.0, 25 °C, 4 h) in each independent experiment, and the ratio of derivative to unmodified-chitin uptake [1].

Experiment	Amine-chitin	Alcohol-chitin	Unmodified chitin	Amine/chitin	Alcohol/chitin
Contact time, 4 h	28.3 ± 2.4	19.1 ± 2.2	5.4 ± 2.1	5.2	3.5
Adsorbent dose, ~1700 mg L^{-1}	31.4 ± 3.0	23.4 ± 2.8	6.3 ± 2.1	5.0	3.7
Isotherm, C_0 = 200 mg L^{-1}	30.9 ± 3.5	23.5 ± 1.1	6.9 ± 1.5	4.5	3.4
pH series, pH 6.0	29.1 ± 1.9	22.0 ± 3.1	6.1 ± 0.8	4.8	3.6
Regeneration, cycle 0	30.8 ± 0.4	23.4 ± 0.7	—	—	—
**Mean across experiments**	**30.1 ± 1.3**	**22.3 ± 1.9**	**6.2 ± 0.6**	**4.9 ± 0.3**	**3.6 ± 0.1**

[1] Each experiment value is the mean ± SD of triplicate independent vessels. The contact-time experiment used 85.0 mg in 50.0 mL; all others used 25.0 mg in 15.0 mL. The last row gives the mean ± SD across experiments (n = 5 for the derivatives, n = 4 for unmodified chitin) and the mean ± SD of the four experiment-wise ratios. These are the values reported in the Abstract and Conclusions.

Table S2. Isotherm parameters with 95% confidence intervals (unweighted nonlinear least squares, as in Table 2) and the corresponding fits weighted by the replicate standard deviations ($1/SD^2$).

Adsorbent	Model	Parameter	Unweighted estimate ± SE	95% CI	Weighted estimate ± SE	Reduced χ^2 (weighted)
Amine-chitin	Langmuir	Q_{max} (mg g^{-1})	37.4 ± 0.8	35.5–39.3	37.1 ± 1.9	0.11
		b (L mg^{-1})	0.0309 ± 0.0024	0.0247–0.0371	0.0317 ± 0.0064	
	Freundlich	K_F	5.7 ± 1.5	1.9–9.5	5.0 ± 0.9	2.27
		n	3.0 ± 0.5	1.8–4.3	2.84 ± 0.29	
Alcohol-chitin	Langmuir	Q_{max} (mg g^{-1})	25.7 ± 0.2	25.2–26.2	25.8 ± 1.3	0.02
		b (L mg^{-1})	0.0683 ± 0.0026	0.0615–0.0750	0.0695 ± 0.0238	
	Freundlich	K_F	7.6 ± 1.5	3.8–11.4	8.7 ± 1.9	0.98
		n	4.6 ± 0.8	2.4–6.7	5.02 ± 1.12	

Seven initial concentrations (25–300 mg L^{-1}), 25.0 mg of adsorbent in 15.0 mL, pH 6.0, 25 °C, 4 h contact; $C_e = C_0 - Q_e \cdot m/V$. 95% CI = estimate ± $t_{0.975,5}$·SE. The weighted fits give the same parameters within uncertainty and the same model ranking; the reduced χ^2 values near or below 1 for the Langmuir model and above 1 for the Freundlich model indicate that only the former describes the data within the replicate scatter.

Table S3. Cu(II) uptake (mg g^{-1}, mean ± SD of three independent vessels) as a function of contact time (Figure 12). Conditions: 85.0 mg of adsorbent in 50.0 mL of 200 mg L^{-1} Cu(II), pH 6.0, 25 °C, 250 rpm.

Time (min)	Amine-chitin	Alcohol-chitin	Unmodified chitin
30	7.6 ± 1.8	4.6 ± 1.0	0.85 ± 0.80
60	13.2 ± 1.2	9.4 ± 1.2	2.57 ± 1.36
90	16.6 ± 1.3	12.5 ± 2.1	3.17 ± 1.41
120	19.7 ± 2.2	14.4 ± 1.1	3.99 ± 1.06
150	23.1 ± 3.2	15.8 ± 1.2	4.82 ± 1.10
180	25.3 ± 1.3	16.8 ± 2.2	5.18 ± 1.78
240	28.3 ± 2.4	19.1 ± 2.2	5.43 ± 2.09
300	29.0 ± 1.3	19.6 ± 2.2	5.54 ± 1.07
1440 (24 h) [1]	30.8 ± 3.1	20.9 ± 3.4	6.5 ± 1.4
2160 (36 h) [1]	36.6 ± 6.1	24.3 ± 5.1	5.6 ± 2.2

[1] Extended-contact experiment of the same design (Section 2.4). None of the extended-contact values differs significantly from the corresponding 240 min value (Welch's t-test, $p = 0.13–0.93$).

Table S4. Cu(II) uptake (mg g^{-1}, mean ± SD, n = 3) as a function of solution pH (Figure 9). Conditions: 25.0 mg of adsorbent in 15.0 mL of 200 mg L^{-1} Cu(II), 25 °C, 4 h.

pH	Amine-chitin	Alcohol-chitin	Unmodified chitin	Adsorbent-free control
3.0	0.48 ± 0.38	0.60 ± 0.55	0.35 ± 0.33	NA
4.0	5.0 ± 1.7	5.6 ± 1.2	1.2 ± 1.0	NA
5.0	20.3 ± 2.5	14.4 ± 1.2	4.3 ± 1.2	NA
6.0	29.1 ± 1.9	22.0 ± 3.1	6.1 ± 0.8	≈3% of dissolved Cu(II) lost over 4 h
7.0	not measured	not measured	not measured	visible precipitate

The ≈3% loss in the adsorbent-free controls at pH 6.0 corresponds to at most 3.6 mg g^{-1} of apparent uptake at the standard dose (Section 3.3). Uptakes are reported without correction.

Table S5. Cu(II) uptake (mg g^{-1}, mean ± SD, n = 3) and percentage of Cu(II) removed as a function of adsorbent dose (Figure 8). Conditions: 200 mg L^{-1} Cu(II), 15.0 mL, pH 6.0, 25 °C, 4 h.

Dose (mg L^{-1})	Amine-chitin uptake	Removal (%)	Dose (mg L^{-1})	Alcohol-chitin uptake	Removal (%)	Dose (mg L^{-1})	Chitin uptake	Removal (%)
100	9.0 ± 1.0	0.5	100	3.0 ± 1.1	0.1	87	0.9 ± 0.90	0.0
334	15.0 ± 2.2	2.5	369	10.0 ± 1.3	1.8	365	1.3 ± 0.84	0.2
695	20.0 ± 1.5	7.0	679	16.0 ± 3.0	5.4	691	3.6 ± 2.20	1.2
1339	28.6 ± 2.6	19.2	1329	20.6 ± 2.7	13.7	1354	4.8 ± 2.10	3.2
1667	31.4 ± 3.0	26.2	1711	23.4 ± 2.8	20.0	1675	6.3 ± 2.10	5.3
2028	28.7 ± 1.1	29.1	1999	20.5 ± 2.4	20.5	1984	5.9 ± 1.50	5.9
2759	26.4 ± 1.1	36.4	2685	16.0 ± 1.0	21.5	2664	5.9 ± 1.00	7.9

Doses are the adsorbent masses actually weighed divided by 15.0 mL. Removal (%) = $Q_e \cdot dose/C_0 \times 100$.

Table S6. Cu(II) uptake (mg g^{-1}, mean ± SD, n = 3) in the initial adsorption (cycle 0) and after each regeneration with 7% (v/v) HCl, and capacity retention relative to cycle 0 (Figure 13). Uptake is normalized to the adsorbent mass recovered at the end of the preceding cycle.

Cycle	Amine-chitin uptake	Retention (%)	Alcohol-chitin uptake	Retention (%)
0	30.8 ± 0.4	100	23.4 ± 0.7	100
1	24.9 ± 1.1	81 ± 4	17.1 ± 1.4	73 ± 6
2	21.4 ± 1.8	69 ± 6	15.5 ± 1.5	66 ± 7
3	17.2 ± 2.9	56 ± 9	12.1 ± 3.1	52 ± 13
4	14.0 ± 3.6	46 ± 12	9.8 ± 4.0	42 ± 17

Retention uncertainties are propagated from the replicate standard deviations of cycles 0 and n. Cycle 4 versus cycle 0: $p = 0.014$ (amine-chitin) and 0.025 (alcohol-chitin), Welch's t-test. Geometric mean loss per cycle: 18% and 20%. Desorption efficiency was not measured.

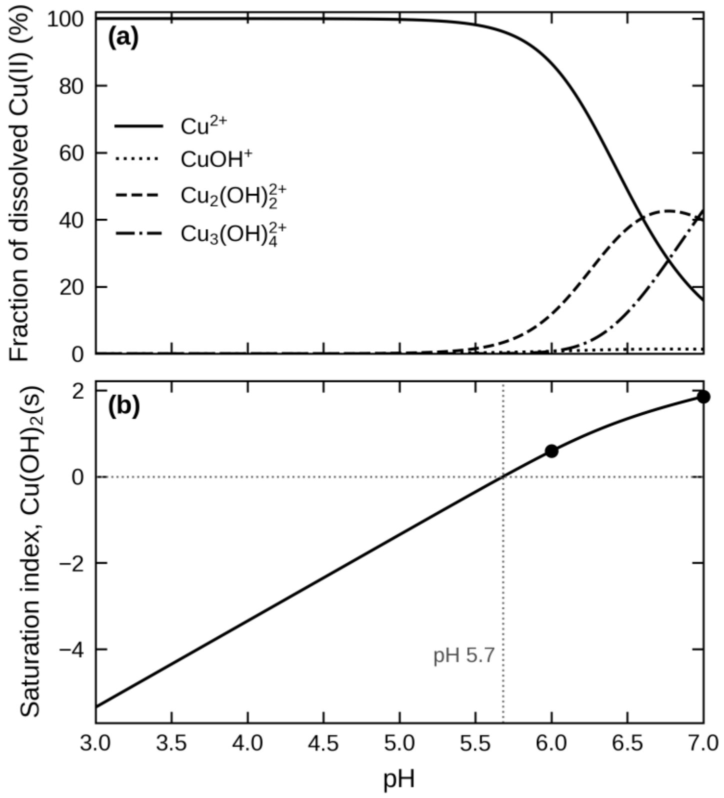

Figure S1. Calculated speciation of 200 mg L^{-1} Cu(II) in nitrate solution at 25 °C (I ≈ 0.01 M) as a function of pH: (**a**) distribution of dissolved Cu(II) species and (**b**) saturation index with respect to $Cu(OH)_2$(s). Calculated from the recommended constants of Powell et al. [53] with Davies activity corrections, for a homogeneous solution without precipitation; species contributing less than 0.5% are not shown. Dotted lines in (**b**) mark saturation (pH ≈ 5.7); filled circles mark pH 6.0 and 7.0.

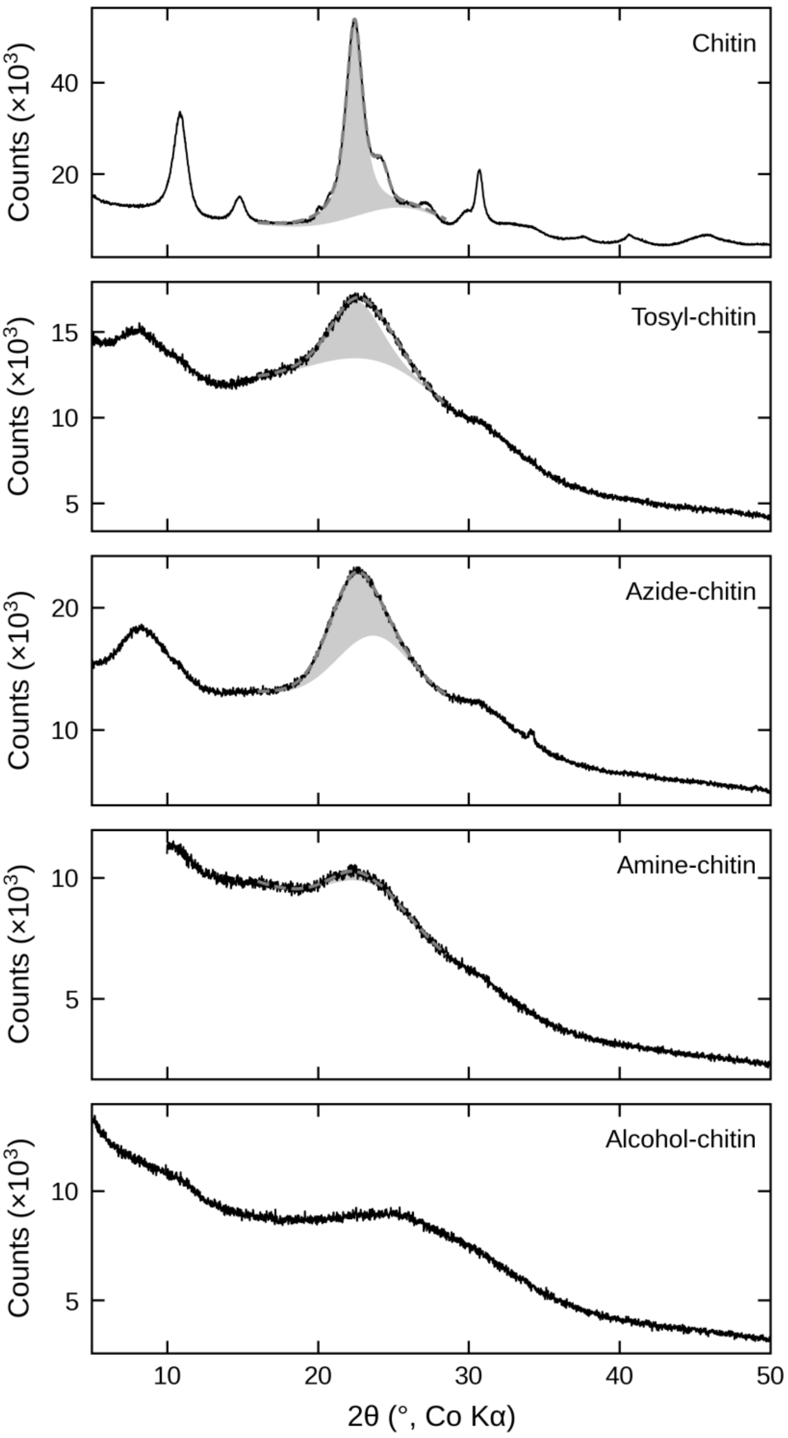

Figure S2. Full X-ray diffraction patterns (Co Kα) of unmodified chitin, tosyl-chitin, azide-chitin, amine-chitin, and alcohol-chitin. Dashed lines show the model fitted over 2θ = 16–28.5°—pseudo-Voigt (110) and neighbouring reflections, a broad amorphous contribution, and a linear background—and the shaded area shows the fitted (110) component used for Table 1. No (110) component could be fitted for alcohol-chitin.
